# Agronomical, Physiological and Biochemical Characterization of In Vitro Selected Eggplant Somaclonal Variants under NaCl Stress

**DOI:** 10.3390/plants10112544

**Published:** 2021-11-22

**Authors:** Sami Hannachi, Stefaan Werbrouck, Insaf Bahrini, Abdelmuhsin Abdelgadir, Hira Affan Siddiqui

**Affiliations:** 1 Department of Biology, College of Science, University of Hail, P.O. Box 2440, Ha’il 81451, Saudi Arabia; nsafafa@yahoo.fr (I.B.); a.abdelmuhsin@uoh.edu.sa (A.A.); 2 Department of Plants and Crops, Faculty of Bioscience Engineering, Ghent University, Coupure Links, 653, 9000 Ghent, Belgium; stefaan.werbrouck@ugent.be; 3 Department of Physics, College of Science, University of Hail, P.O. Box 2440, Ha’il 81451, Saudi Arabia; ha.siddiqui@uoh.edu.sa

**Keywords:** salt tolerance, eggplant, somaclonal variants, growth, proline, MDA carbohydrates, photosystem II, sodium, chloride

## Abstract

Previously, an efficient regeneration protocol was established and applied to regenerate plants from calli lines that could grow on eggplant leaf explants after a stepwise in vitro selection for tolerance to salt stress. Plants were regenerated from calli lines that could tolerate up to 120 mM NaCl. For further in vitro and in vivo evaluation, four plants with a higher number of leaves and longer roots were selected from the 32 plants tested in vitro. The aim of this study was to confirm the stability of salt tolerance in the progeny of these four mutants (‘R18’, ‘R19’, ‘R23’ and ‘R30’). After three years of in vivo culture, we evaluated the impact of NaCl stress on agronomic, physiological and biochemical parameters compared to the parental control (‘P’). The regenerated and control plants were assessed under in vitro and in vivo conditions and were subjected to 0, 40, 80 and 160 mM of NaCl. Our results show significant variation in salinity tolerance among regenerated and control plants, indicating the superiority of four regenerants (‘R18’, ‘R19’, ‘R23’ and ‘R30’) when compared to the parental line (‘P’). In vitro germination kinetics and young seedling growth divided the lines into a sensitive and a tolerant group. ‘P’ tolerate only moderate salt stress, up to 40 mM NaCl, while the tolerance level of ‘R18’, ‘R19’, ‘R23’ and ‘R30’ was up to 80 mM NaCl. The quantum yield of PSII (Φ_PSII_) declined significantly in ‘P’ under salt stress. The photochemical quenching was reduced while nonphotochemical quenching rose in ‘P’ under salt stress. Interestingly, the regenerants (‘R18’, ‘R19’, ‘R23’ and ‘R30’) exhibited high apparent salt tolerance by maintaining quite stable Chl fluorescence parameters. Rising NaCl concentration led to a substantial increase in foliar proline, malondialdehyde and soluble carbohydrates accumulation in ‘P’. On the contrary, ‘R18’, ‘R19’, ‘R23’ and ‘R30’ exhibited a decline in soluble carbohydrates and a significant enhancement in starch under salinity conditions. The water status reflected by midday leaf water potential (ψl) and leaf osmotic potential (ψπ) was significantly affected in ‘P’ and was maintained a stable level in ‘R18’, ‘R19’, ‘R23’ and ‘R30’ under salt stress. The increase in foliar Na^+^ and Cl^−^ content was more accentuated in parental plants than in regenerated plants. The leaf K^+^, Ca^2+^ and Mg^2+^ content reduction was more aggravated under salt stress in ‘P’. Under increased salt concentration, ‘R18’, ‘R19’, ‘R23’ and ‘R30’ associate lower foliar Na^+^ content with a higher plant tolerance index (PTI), thus maintaining a normal growth, while foliar Na^+^ accumulation was more pronounced in ‘P’, revealing their failure in maintaining normal growth under salinity stress. ‘R18’, ‘R19’, ‘R23’ and ‘R30’ showed an obvious salt tolerance by maintaining significantly high chlorophyll content. In ‘R18’, ‘R19’, ‘R23’ and ‘R30’, the enzyme scavenging machinery was more performant in the roots compared to the leaves. Salt stress led to a significant augmentation of catalase, ascorbate peroxidase and guaiacol peroxidase activities in the roots of ‘R18’, ‘R19’, ‘R23’ and ‘R30’. In contrast, enzyme activities were less enhanced in ‘P’, indicating lower efficiency to cope with oxidative stress than in ‘R18’, ‘R19’, ‘R23’ and ‘R30’. ACC deaminase activity was significantly higher in ‘R18’, ‘R19’, ‘R23’ and ‘R30’ than in ‘P’. The present study suggests that regenerated plants ‘R18’, ‘R19’, ‘R23’ and ‘R30’ showed an evident stability in tolerating salinity, which shows their potential to be adopted as interesting selected mutants, providing the desired salt tolerance trait in eggplant.

## 1. Introduction

Soil salinity is a major abiotic factor, influencing over 1000 million ha of land in several parts of the world, and is one of the main current constraints in developing irrigated agriculture [1,2]. An excess of salt causes several physiological and biochemical problems such as ion imbalance, osmotic stress, ion toxicity and oxidative stress [3], thus reducing the growth and productivity of numerous crops [4,5]. In vitro culture, as an induced somaclonal variation, proved to be a promising and effective method to obtain salt-tolerant genotypes. The selection of salt-tolerant cell lines has been reported for numerous crops [6,7]. However, the results are not always predictable. The tolerance of cell lines is often transient, rather than a stable and heritable adaptation [8,9]. Therefore, the offspring of somaclonal variants should be further screened and selected under field conditions to confirm the stability of the salt tolerance obtained [10,11]. Somaclonal variation can be evaluated not only phenotypically, but also through the number of chromosomes, chromosome structure, proteins or direct DNA analysis [12,13,14].

The investigation of salt tolerance during different growth stages is important for elucidating saline limits at each developmental phase [15]. Salt stress reduced and delayed the germination of solanaceous vegetables such as tomatoes [16] and eggplant [17,18]. The reduced seed germination induced by salinity could be caused by the induction of dormancy, by osmotic stress or by specific ion toxicity [19].

Salts influence plant growth by increasing the osmotic pressure in the soil and interfering with plant nutrition [20]. Carbohydrates, which are required for cell growth, are supplied mainly through the photosynthesis process, and the photosynthesis rates are usually lower in plants exposed to salt and especially to NaCl. The accumulation of compatible solutes, such as proline and polyols, in the cytoplasm is required to compensate for the decrease in water potential occurring in the vacuole due to ion accumulation in that compartment [21]. Commonly used biochemical markers of abiotic stress, including salinity, are ROS and lipid peroxidation products [22].

Abiotic stress contributes to the production of reactive oxygen species (ROS) in plants. ROS are continuously produced in chloroplasts, mitochondria and peroxisomes in higher plants [23]. However, H_2_O_2_ produced in peroxisomes and chloroplasts diffuses to the cytosol and is converted to hydroxyl radicals by the Fenton reaction [24]. The overproduction of ROS (such as superoxide (O_2_^−^), singlet oxygen (O_2_), hydrogen peroxide (H_2_O_2_) and hydroxyl radicals (OH^−^)) in plant cells under stress contributes to the damage of cellular components, including DNA, proteins and membrane lipids, and leads to the destruction of photosynthetic pigments [25,26]. Abiotic stress may induce oxidative stress in plants and generates cellular adaptive responses such as the induction of stress proteins and acceleration of ROS-scavenging systems [3]. According to [27], to scavenge reactive oxygen species, plants have evolved both enzymatic and non-enzymatic defense systems. The enzymatic antioxidants including superoxide dismutase (SOD), catalase (CAT), ascorbate peroxidase (APX), guaiacol peroxidase (POD) and glutathione reductase (GR) [28]. The authors in [29] showed that ROS is converted by SOD into H_2_O_2_, which is further scavenged by CAT and various peroxidases (APX, POD) to H_2_O.

On the other hand, salinity tolerance usually correlates with high amounts of compatible solutes and efficient compartmentation, as shown in a large set of studies [30]. Both salt tolerance and the sensitivity of a specific crop depend on its ability to extract water and nutrients from saline soils and to avoid excessive tissue accumulation of salt ions [31,32,33]. The ion toxicities have diverse consequences resulting in ionic imbalance, i.e., in terms of the uptake competition of Na^+^ with K^+^, Ca^2+^ and Mg^2+^, and may reduce the availability of the beneficial nutrients [34].

Salinity also affects photosynthesis mainly through a reduction in leaf area, chlorophyll content and stomatal conductance, and to a lesser extent through a decrease in photosystem II efficiency [35].

Additionally, in susceptible tomato cultivars [36] chlorophyll fluorescence measurements showed a considerable decrease in the efficiency of PSII and ETR under increasing salt stress. The absorbed energy non-utilized in the photochemical pathway was dissipated as heat and this was confirmed by the concomitant increase in NPQ. Such an increase was suggested to cause a downregulation of PSII in order to avoid an over-reduction in Q_A._

Plants develop several strategies to overcome NaCl stress, including the plant growth-promoting bacteria (PGPB) living in the rhizosphere and rhizoplane of the plants [37,38,39]. The PGPB involve several beneficial bacteria which contribute efficiently to increasing plant salt tolerance by decreasing the ethylene level through hydrolysis of 1-aminocyclopropane-1-carboxylic acid (ACC) by the ACC deaminase enzyme and convert it into ammonia and α-ketobutyrate. ACC is the direct precursor of the hormone ethylene in plants [40,41]. Consequently, the PGPR with their ACC deaminase activity lower the abiotic stress engendered by ethylene synthesis and its impact on plants [42,43,44,45]. 

Eggplant (*Solanum melongena* L.) is one of the most popular and economically important vegetable crops worldwide [46]. It is considered to be moderately sensitive to salinity [47]. Therefore, improving the salt tolerance of the current existing eggplant germplasm seems to be of immense importance in order to increase crop productivity. In several crops, spontaneous or induced somaclonal variation induced during the in vitro culture of plant cells occurs as a promising strategy for obtaining salt-tolerant mutants [48,49,50]. However, the assessment of the genetic stability of salt-tolerant selected somaclones is highly required [51,52,53]. Although successful eggplant somatic embryogenesis [53,54] and shoot organogenesis [55,56] was reported, the first attempt to produce and select a salt-tolerant eggplant cell line was only achieved recently. 

In the present work, we analyzed the progeny of eggplant somaclonal variants, which had been obtained previously by a stepwise increasing salinity (40, 80, 120 or 160 mM NaCl) in vitro selection and screened for salt tolerance [57]. These somaclones were regenerated on the calli lines that could tolerate up to 120 mM NaCl. From 32 plants tested in vitro, four plants with a superior number of leaves and root length were selected for further in vitro and in vivo evaluation. 

The present study aimed to identify possible stable salt-tolerant genotypes among the progeny of somaclonal variants and characterize them through agronomic, physiological and biochemical parameters.

## 2. Results

We analyzed the progeny of selected eggplant somaclonal variants, which had been obtained previously by a stepwise increasing salinity (40, 80, 120 or 160 mM NaCl) in vitro selection and screened for salt tolerance. These somaclones were regenerated on the calli lines that could tolerate up to 120 mM NaCl. Out of thirty two plants tested in vitro, four plants with a higher number of leaves and root length were used for further in vitro and in vivo evaluation.

### 2.1. Salinity-Induced Change in Seed Germination Parameters (Exp 1)

Although it does not fully reproduce the field behaviour of plants, the germination kinetics under controlled saline conditions provide a trend about the differential behaviour of the studied cultivars in response to the applied stress.

**Figure 1:**
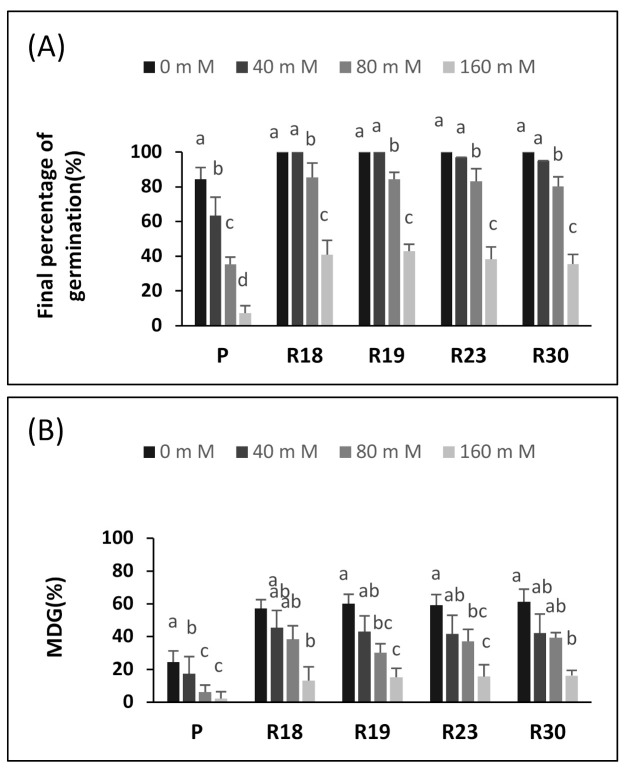

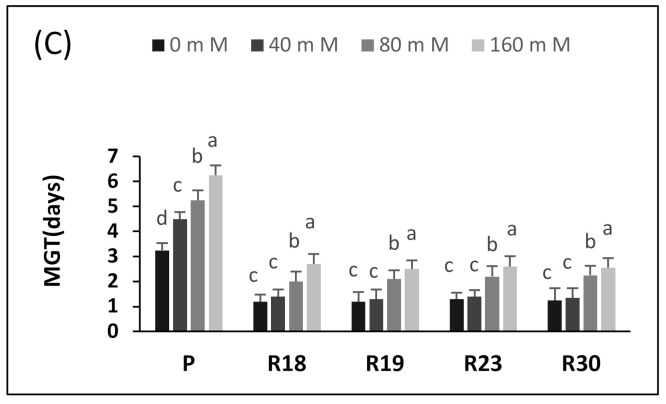
Effect of increasing NaCl on the FG (%) (**A**), MDG (%) (**B**) and MGT (days) (**C**). Comparisons between means were made with Tukey’s HSD test (*p* = 0.05). Values are means ± SE (*n* = 5).

We studied the impact of increasing NaCl on germination kinetics as assessed by the final germination (FG,%), mean daily germination (MDG, %) and mean germination time (MGT, days) of the parent and regenerant lines (Figure 1).

In the control treatment, FG was high, reaching 84.3% in the parental control (‘P’) and 100% in all regenerants (‘R18’, ‘R19’, ‘R23’ and ‘R30’). A salt concentration of 40 mM significantly reduced FG in ‘P’ (63.5%) but had little effect on ‘R18’ (100%), ‘R19’ (100%), ‘R23’ (97%) and ‘R30’ (95%). When the salt concentration increased, the FG decreased significantly. The highest NaCl concentration (160 mM) significantly reduced FG in all lines evaluated. This reduction was clearly stronger in ‘P’ (91.7%) than in ‘R18’ (60%), ‘R19’ (57.1%), ‘R23’ (61.1%) and in ‘R30’ (64.5%) (Figure 1A).

A salt concentration of 40 mM NaCl already reduced MDG in ‘P’ (Figure 1B). A higher salt concentration (80 mM NaCl) caused a significant decline in MDG in ‘P’, ‘R18’, ‘R19’, ‘R23’ and ‘R30’ by 74.1%, 33.3%, 50%, 37% and 36%, respectively, compared to controls. Although the highest salinity level decreased the MDG significantly in all lines, ‘P’ exhibited the most pronounced reduction (90.8%) (Figure 1B). 

MGT is a measure of the rapidity of germination, with lower values indicating faster germination. The evaluation of the impact of salinity on the MGT showed a clear difference between the parental and regenerated lines. The first dissimilarity was observed in the control treatment, showing that germination was faster in ‘R18’, ‘R19’, ‘R23’ and ‘R30’ than in ‘P’. The higher salt concentration increased MGT significantly from 80 mM NaCl on. At 160 mM NaCl, MGT doubled in all lines compared to the controls (Figure 1C).

### 2.2. Salinity-Induced Change in In Vitro Seedling Growth (Exp 1)

The effect of increasing NaCl concentration on parent and regenerant growth parameters, as evaluated by plant height (PH), leaf number (LN), aerial fresh weight (FW), aerial dry weight (DW) and tissue water content (TWC) of eggplant seedlings after 45 days of growth, is shown in Table 1.

Increasing salinity level had no effect on LN in ‘R18’, ‘R19’, ‘R23’ and ‘R30’ but decreased the same parameter significantly in ‘P’ by 24% and 68% at 40 mM and 80 mM NaCl, respectively, compared to controls. However, PH was decreased for 80 mM NaCl in ‘P’, ‘R18’, ‘R19’, ‘R23’ and ‘R30’ with a reduction of 60%, 55%, 50%, 34.5% and 46%, respectively. For the parental control, LN declined from 40 mM NaCl and PH was reduced from 80 mM on (Table 1). 

The FW of all lines decreased as the salinity increased (Table 1). Although plant vigor differed among the parents and regenerants as indicated by their FW, the salt-induced declines in FW showed a similar overall trend. The maximum decrease in the FW was observed in ‘P’. As compared with the control conditions, the FW was reduced by 94.5% in ‘P’, at 80 mM NaCl (Table 1). In contrast, the decline in the FW under 80 mM NaCl was 32.7%, 34.5%, 37.7% and 40% in ‘R18’, ‘R19’, ‘R23’ and ‘R30’, respectively (Table 1).

Under salinity conditions, DW declined in all lines. The most important decline was found in ‘P’ by 78.3% for 80 mM NaCl (Table 1). On the contrary, at 80 mM NaCl, ‘R18’, ‘R19’, ‘R23’ and ‘R30’ exhibited a lower reduction in the DW, reaching 35.3%, 35%, 40% and 40%, respectively, compared to controls (Table 1). Moreover, the parental control showed a substantial decrease in TWC at the highest salinity level (80 mM NaCl) compared to controls. In contrast, regenerants succeeded in keeping their TWC quite steady for the investigated salt concentrations (Table 1).

### 2.3. NaCl-Induced Changes in Plant–Water Relations, Growth and Yield (Exp 2)

#### 2.3.1. Plant–Water Relations

**Figure 2:**
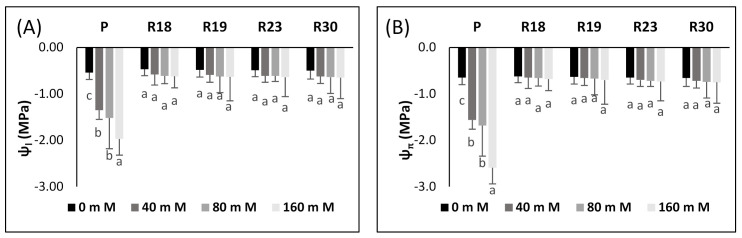
Effect of increasing levels of NaCl on ψl (**A**) and ψπ (**B**) of parents and regenerants. Comparisons between means were made by Tukey’s HSD test (*p* = 0.05). Values are means ± SE (*n* = 5).

The impact of increasing NaCl levels on plant–water relations, as assessed by the midday leaf water potential (ψl) and leaf osmotic potential (ψπ) of parents and regenerants, is given in Figure 2.

Increasing salinity level significantly reduced midday Ψ_l_ in the parental control and attained −1.97 MPa after 30 days at the highest salt stress concentration (160 mM NaCl) (Figure 1A). In contrast, all the regenerated plants succeeded in keeping their Ψ_l_ quite stable under increasing salinity levels. The recorded values of Ψ_l_ at 160 mM NaCl in ‘R18’, ‘R19’, ‘R23’ and ‘R30’ ranged from −0.62 to −0.65 MPa. Therefore, the regenerated plants were hardly affected by osmotic stress (Figure 2A).

Ψ_π_ showed a similar trend to Ψ_l_ under salinity conditions. After 30 days of salt stress application, a salt concentration of 160 mM NaCl decreased Ψ_π_ significantly in ‘P’ and attained −2.59 MPa. On the contrary ‘R18’, ‘R19’, ‘R23’ and ‘R30’ were able to maintain their Ψ_π_ at a more stable level (Figure 2A,B) 

#### 2.3.2. Plant Growth and Yield Evaluation (Exp 2)

The impact of increasing NaCl levels on plant growth, as investigated by aerial fresh weight (FW), aerial dry weight (DW) and tissue water content (TWC) of parents and regenerants, is shown in Table 2, Table 3 and Table 4.

All salinity levels contributed to a significantly higher shoots biomass in the regenerated plants when compared to the control parental line (Table 2). Indeed, ‘R18’, ‘R19’, ‘R23’ and ‘R30’ maintained significantly higher FW and DW than ‘P’ (Table 2 and Table 3). The TWC was significantly greater in all regenerated plants compared to the parental control in all treatments. In addition, the highest salt concentration caused TWC to be reduced significantly in ‘P’. However, ‘R18’, ‘R19’, ‘R23’ and ‘R30’ maintained a quite stable TWC regardless of the salinity level (Table 4). 

The impact of salt stress on the yield parameters (fruit number per plant and mean fresh weight per fruit) of parents and regenerants subjected to an increasing salt stress level for five weeks and harvested 45 days after flowering is given in Table 5.

There was a significant difference in the number of fruits observed between the regenerants and the parental line. In the absence of salt stress, all regenerated plants produced more fruit than the parental line. Compared to the parental line, all plants produced from somaclonal lines also yielded more fruit at a salt concentration up to 80 mM NaCl, with the increase ranging from 225% (‘R18’, ‘R19’, ‘R30’) to 255% (‘R23’) (Table 5). At 120 mM NaCl, the fruit yield decreased in all plants, compared to 80 mM NaCl. Nevertheless, this decrease was less pronounced, particularly in the regenerants ‘R18’ (15.3%), ‘R19’ (15.3%), ‘R23’ (14.3%) and ‘R30’ (23%) than in the parental line (75%) (Table 5). The same trend was observed with regard to the fruit weight, showing that the decline was more severe in the parental line (47.4%) than in the regenerants ‘R18’ (30.7%), ‘R19’ (23.07%), ‘R23’ (23.2%) and ‘R30’ (21.7%) (Table 5).

#### 2.3.3. NaCl-Induced Changes in Chl Fluorescence Parameters (Exp 2)

**Figure 3:**
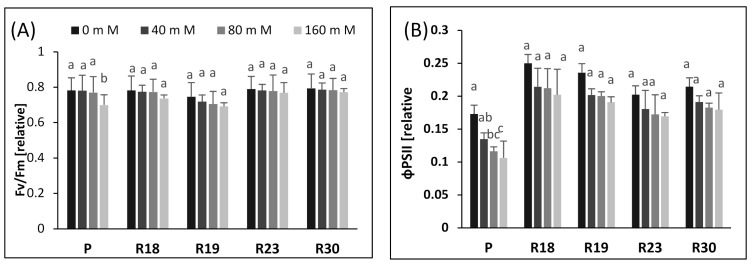

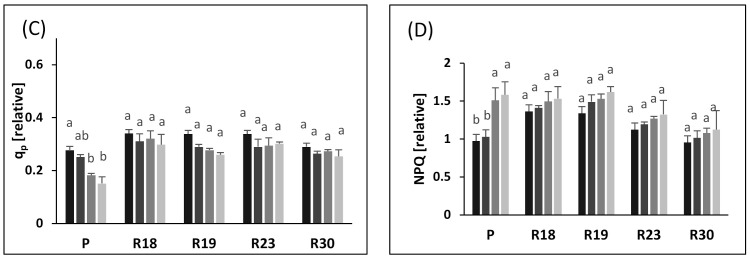
Impact of several salt concentrations (0, 40, 80, and 160 mM) after 30 days under NaCl stress on chlorophyll fluorescence parameters: the maximum quantum yield of PSII (Fv/Fm) (**A**), effective quantum yield of PSII (ΦPSII) (**B**), photochemical quenching (q_p_) (**C**) and non-photochemical quenching (NPQ) (**D**). Different uppercase letters indicate significant differences using Tukey’s test (*p* = 0.05). Data are means of five replicates ± SE.

The effect of different salt concentrations (0, 40, 80, and 160 mM) after 30 days under NaCl stress on the chlorophyll fluorescence parameters of parents and regenerants (the maximum quantum yield of PSII, effective quantum yield of PSII, photochemical quenching and non-photochemical quenching) is shown in Figure 3.

After 15 days, salinity stress did not significantly affect Fv/Fm, qp, or NPQ in the parental control or in the selected regenerated plant in all salinity concentrations (data not shown). After 30 days of salt stress imposition, a significant reduction in F_v_/F_m_ occurred in ‘P’ for the highest salt concentration (160 mM NaCl). However, ‘R18’, ‘R19’, ‘R23’ and ‘R30’ were hardly affected by salinity in all treatments (Figure 3A).

The significant decrease in Φ_PSII_ was found only in ‘P’ after 30 days of salt stress imposition (Figure 3B). A salt concentration of 160 mM NaCl caused Φ_PSII_ to be reduced by 38.7% in ‘P’, compared to the control. In contrast, in ‘R18’, ‘R19’, ‘R23’ and ‘R30’, Φ_PSII_ was hardly affected after 30 days of subjection to salt stress in all treatment (Figure 3B).

q_p_ declined significantly only after 30 days of salt stress application in the parental control (Figure 3C). A salt concentration of 160 mM NaCl generated a decrease of 45.4% of q_p_ in ‘P’ relative to the control. On the contrary, qp reduction was not significant in all regenerated plants for all salinity treatments during the whole experimental period (Figure 3C).

NPQ showed a similar but opposite trend as q_p_ in ‘P’. A salinity level of 160 mM NaCl significantly boosted NPQ by 62.42% in ‘P’ relative to the control, after 30 days of salt stress (Figure 3D). In contrast, salt stress had no significant effect on NPQ in ‘R18’, ‘R19’, ‘R23’ and ‘R30’ after 30 days of salt stress imposition in all treatments (Figure 3D).

#### 2.3.4. NaCl-Induced Changes in Leaf Chlorophyll Content (Exp 2)

The chlorophyll data average of parents and regenerants subjected to increasing salt stress levels during five and ten weeks in the greenhouse is given in Table 6 and Table 7.

The chlorophyll content was hardly affected after the exposure of the parental and regenerated plant lines to increasing salt concentrations for five weeks (Table 6). However, we observed significant differences in chlorophyll content between all regenerants and the parental control lines at each treatment with salt concentration. ‘R18’, ‘R19’, ‘R23’ and ‘R30’ demonstrated their superior salt tolerance with better vegetative growth compared with ‘P’. A significant difference was found in chlorophyll content between the two measurement dates. The regenerated plants showed higher chlorophyll content with increasing salinity compared to the parental control (Table 6 and Table 7). In general, our results show that the onset of salinity stress and its intensification over a long period of time reduced the chlorophyll content. At the highest salt concentration, although a reduction in chlorophyll content was observed in ‘R18’, ‘R19’, ‘R23’ and ‘R30’, it was less pronounced than in ‘P’. Consequently, the regenerated plants showed a high capacity to cope with increasing salinity stress while managing to maintain an increased chlorophyll content. 

### 2.4. NaCl-Induced Changes in Metabolites (Exp 1 and 2)

**Figure 4:**
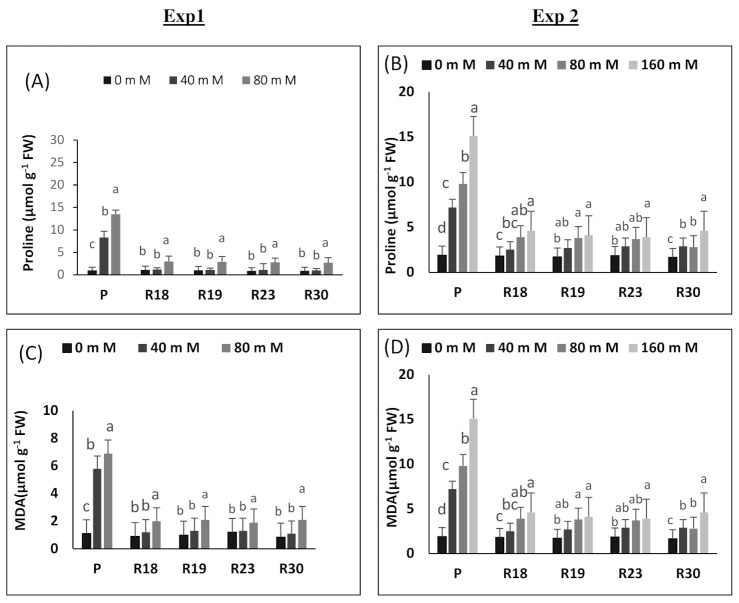
Effect of salt stress on leaf proline content (μmol g^−1^ FW) (**A**,**B**) and on leaf lipid peroxidation (**C**,**D**) of parents and regenerants subjected to different NaCl concentrations. Comparisons between means were made by Tukey’s HSD test (*p* = 0.05). Values are means ± SE (*n* = 5).

Proline and MDA were analyzed in both the in vitro seedlings (Figure 4A,C) and the greenhouse parents and regenerants (Figure 4B,D).

#### 2.4.1. Experiment 1

Leaf proline rose significantly under increasing salinity levels in all studied lines. The highest proline accumulation was found in ‘P’, which exhibited a significant increase at 40 mM NaCl, while in ‘R18’, ‘R19’, ‘R23’ and ‘R30’ a moderate rising was only recorded at the highest salt concentration (80 mM NaCl). In ‘P’, proline accumulation was hugely increased depending on the intensity of salt stress. The strongest increase in proline accumulation was found in ‘P’, at 80 mM NaCl (Figure 4A).

The same trend observed for proline was also found in MDA under saline conditions. In ‘P’, MDA rose 7-fold in the 80 mM NaCl level compared to the control, whereas the MDA increase in ‘R18’, ‘R19’, ‘R23’ and ‘R30’ was, respectively, 2.2-fold, 2.1-fold, 1.6-fold and 2.4-fold for the highest salt level (80 mM NaCl) compared to the respective controls (Figure 4C).

#### 2.4.2. Experiment 2

Increasing salt concentration contributed to a significant rise in proline content in the parental control and regenerated plant lines. However, proline’s increase was less accentuated in ‘R18’, ‘R19’, ‘R23’ and ‘R30’ than in ‘P’. A salinity level of 160 mM NaCl increased proline accumulation, respectively, by 89.1%, 59.01%, 55.7%, 55.5% and 51.6% in ‘P’, ‘R18’, ‘R19’, ‘R23’ and ‘R30’ relative to controls (Figure 4B). 

Although MDA content rose significantly in the parental control and regenerated plant lines, the accumulation was more accentuated in ‘P’ than in ‘R18’, ‘R19’, ‘R23’ and ‘R30’. The highest salt concentration (160 mM NaCl) caused MDA to increase, respectively, 8-fold, 3-fold, 2.4-fold, 2-fold and 2.8-fold in ‘P’, ‘R18’, ‘R19’, ‘R23’ and ‘R30’ compared to controls. Therefore, the lipid peroxidation level was higher in the parental control than in the regenerated plants under salinity conditions (Figure 4D). It is noteworthy that leaf proline accumulation and lipid peroxidation were more pronounced in the greenhouse experiment than in the in vitro experiment.

**Figure 5:**
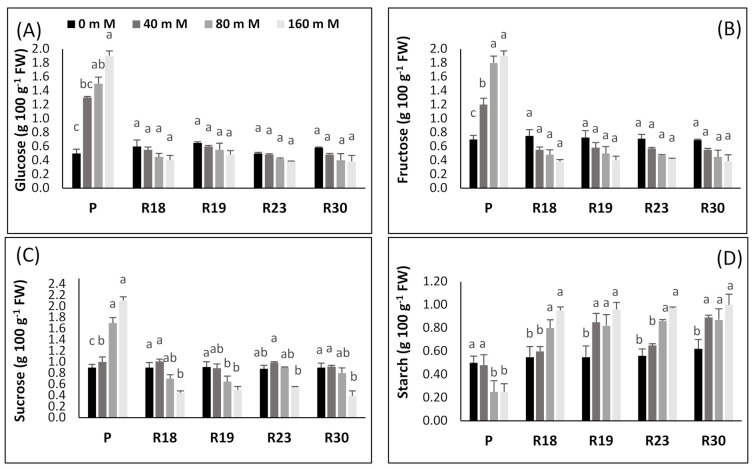
Effect of NaCl concentration on glucose (**A**), fructose (**B**), sucrose (**C**) and starch (**D**) levels in leaves of parents and regenerants. Comparisons between means were made by Tukey’s HSD test (*p* = 0.05). Values are means ± SE (*n* = 5).

The impact of NaCl concentration on carbohydrates as assessed by glucose, fructose, sucrose, and starch levels in the leaves of parents and regenerants is given in Figure 5.

The parental control line exhibited a significant elevation in foliar glucose, fructose and sucrose content under increasing salinity (Figure 5A–C). In contrast, regenerated plants showed a non-significant decline in foliar glucose and fructose content and a significant decline in leaf sucrose content as salt concentration rose (Figure 5A–C). 

Salt stress augmentation generated a significant rise in starch content in ‘R18’, ‘R19’, ‘R23’ and ‘R30’; however, there was significantly less starch piling up in ‘P’ (Figure 5D).

### 2.5. NaCl-Induced Changes in Mineral Content (Exp 2)

The effect of NaCl salinity on the accumulation of K, Ca, Mg, Na, P, NO_3_^−^, Cl^−^ and on Na/K and Na/Ca ratios in control parent and regenerant leaves is shown in Table 8. 

Increasing salinity concentration boosted leaf Na^+^ and Cl^-^ accumulation in parent and all regenerants (Table 8). These dissimilarities were significant from 40 mM NaCl in P and from 80 mM NaCl on in ‘R18’, ‘R19’, ‘R23’ and ‘R30’. It is remarkable that the most pronounced rise was exhibited by the parental control for the highest salt level (160 mM NaCl). Indeed, leaf Na accumulation in ‘P’ was stronger and was 2.73-fold, 2.8-fold, 2.4-fold and 2.5-fold higher than Na concentration in ‘R18’, ‘R19’, ‘R23’ and ‘R30’ leaves, respectively (Table 8).

Leaf K^+^ content declined significantly from 80 mM NaCl for the control parental line and from 160 mM NaCl for the regenerant lines (Table 8). Interestingly, the reduction in leaf K^+^ concentration for ‘R18’ (24.1%), ‘R19’ (23.7%), ‘R23’ (30%) and ‘R30’ (25.8%) was less accentuated than for ‘P’ (42.1%) when compared to their respective controls (Table 8). 

Rising salinity level generated no significant effect on Ca^2+^ accumulation in all regenerated plants. In contrast, a salt concentration of 160 mM NaCl caused Ca^2+^ content to be significantly reduced in the control parental line (Table 8). 

Foliar Mg concentration was hardly affected by rising salt stress in ‘R18’, ‘R19’, and ‘R30’, whereas the highest salinity level (160 mM NaCl) decreased Mg content in ‘P’ and ‘R23’ (Table 8). 

Although foliar Na^+^/K^+^ and Na^+^/Ca^2+^ ratios rose significantly by increasing salinity for the parental control and regenerated plants, it was evident that these ratios were higher in ‘P’ than in ‘R18’, ‘R19’, ‘R23’ and ‘R30’, for all salinity treatments (Table 8). 

Furthermore, salt stress affected nitrate concentration in all lines. However, the reduction was only significant in the parental control. Indeed, foliar nitrate declined from 80 mM NaCl in ‘P’, whereas no significant impact on leaf nitrate concentration was noticed in ‘R18’, ‘R19’, ‘R23’ and ‘R30’.

### 2.6. NaCl-Induced Changes in Enzyme Activities (Exp 2)

**Figure 6:**
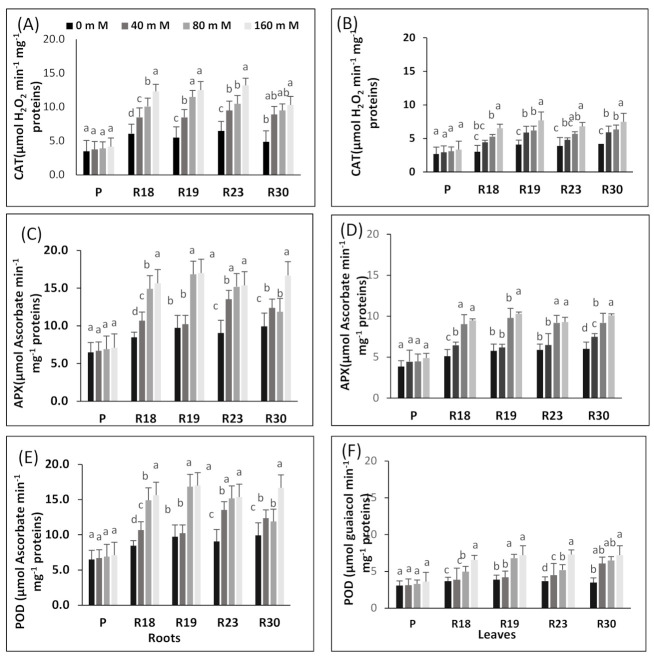
Antioxidant enzyme responses to NaCl treatments in parents and regenerants. Catalase (CAT) activity in roots (**A**) and in leaves (**B**), Ascorbate peroxidase (APX) activity in roots (**C**) and in leaves (**D**), Guaiacol peroxidase (POD) activity in roots (**E**) and in leaves (**F**). Values are means of five replicates ± SE (*n* = 5). Significant dissimilarities between treatments (*p* = 0.05) based on Tukey’s HSD test are shown by different lowercase letters.

The antioxidant enzyme responses to NaCl treatments in parent and regenerant roots and leaves are shown in Figure 6.

Rising salt concentration generated a significant increase in CAT activity in ‘R18’, ‘R19’, ‘R23’ and ‘R30’ in roots and leaves (Figure 6A,B). Root CAT activity increased 2.1-, 2.2-, 2- and 2.1-fold at 160 mM NaCl, respectively, in ‘R18’, ‘R19’, ‘R23’ and ‘R30’ relative to the control. On the contrary, P exhibited lower root CAT activity and attained only a 1.2-fold increase at 160 mM NaCl relative to the control.

All regenerated plants showed higher CAT activity than the parental control in all salinity treatments (Figure 6A,B). In addition, CAT activity was more enhanced in roots than leaves in ‘P’, ‘R18’, ‘R19’, ‘R23’ and ‘R30’ (Figure 6A,B). 

Similar to CAT activity, root and leaf APX and POD activities rose significantly in ‘R18’, ‘R19’, ‘R23’ and ‘R30’ under salinity conditions (Figure 6C–F). However, the increase in APX and POD activities was more pronounced in roots than in leaves (Figure 6C–F). Increasing salt concentrations boosted root APX activity 1.9-, 1.8-, 1.7- and 1.7-fold at 160 mM NaCl, respectively, in ‘R18’, ‘R19’, ‘R23’ and ‘R30’ compared to control (Figure 6C), whereas APX activity was hardly affected in ‘P’ (Figure 6D). Rising salt concentration increased POD activity 2-, 1.9-, 1.8- and 1.8-fold at 160 mM NaCl, respectively, in ‘R18’, ‘R19’, ‘R23’ and ‘R30’ compared to control (Figure 6E). In contrast, POD activity was hardly changed in roots and leaves in ‘P’ (Figure 6C–F).

### 2.7. Plant Tolerance Index (PTI) of Parents and Regenerants (Exp 2)

A plant tolerance index (PTI) was calculated based on the total fresh weight (FW) in salt-stressed plants per total FW in control plants.

**Figure 7:**
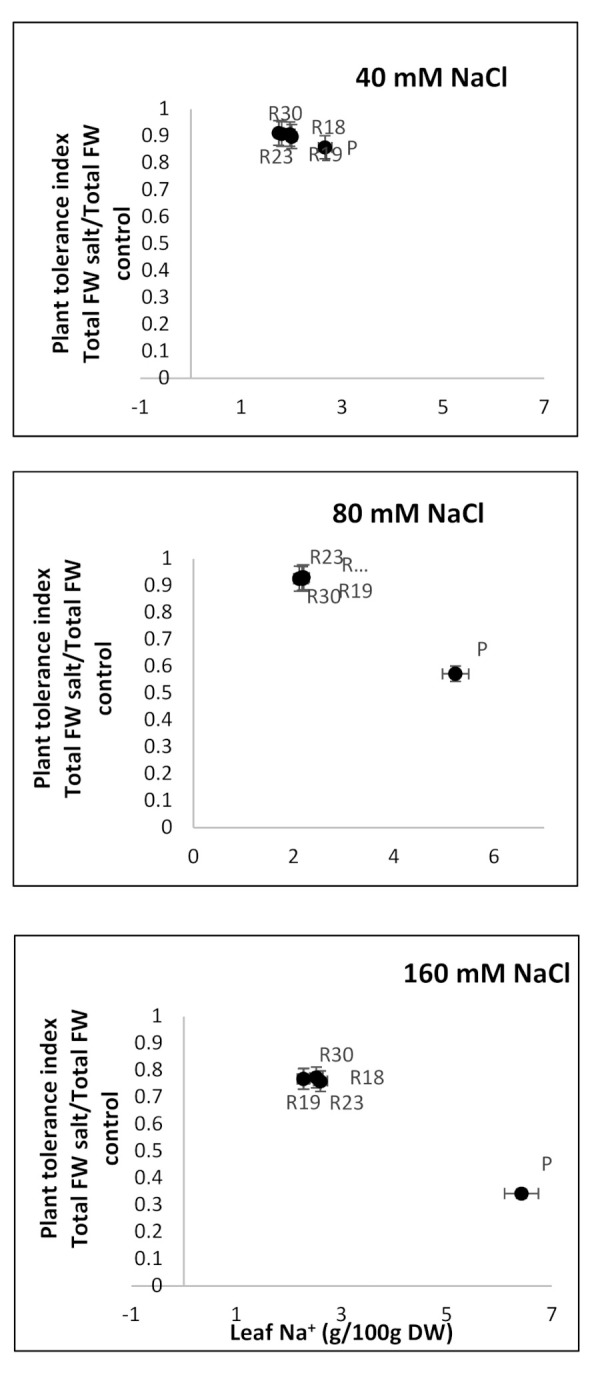
Relationship between leaf sodium content and plant salinity tolerance, as measured by total fresh weight (FW) in salt-stressed plant ÷ total FW in control plant, in parent and somaclones. Values are means of five replicates ± SE (*n* = 5).

The plant tolerance index (PTI) of parental control and somaclonal lines subjected to 40 mM, 80 mM and 160 mM NaCl was estimated and linked to the foliar Na^+^ content. We obtained a significant negative correlation for parental control and all regenerated plants (r = −0.87 for P, r = −0.78 for R18, r = −0.76 for R19, r = −0.77 for R23 and r = −0.78 for R30). While salt concentration rose, ‘R18’, ‘R19’, ‘R23’ and ‘R30’ maintained high PTI and low foliar Na^+^ content, thus contributing to normal growth (Figure 7). In contrast, ‘P’ was unable to maintain a high PTI and exhibited high foliar Na^+^ accumulation, thus generating a low level of growth (Figure 7).

### 2.8. ACC Deaminase-Producing Bacteria Assay (Exp 2)

The deaminase activity of the enzyme 1-aminociclopropane-1-carboxylase (ACC) is one of the key traits used by PGPB to decrease ethylene levels under salt stress. The ACC deaminase converts ACC into ammonia and α-ketobutyrate.

#### 2.8.1. Bacterial Isolation and Preliminary Assessment of ACC Deaminase Activity

Fifteen bacteria were isolated from the roots of ‘P’, ‘R18’, ‘R19’, ‘R23’ and ‘R30’ on enrichment media, of whic, four strains had the capacity of growing on DF minimal salt medium supplemented with 3 mM ACC as a nitrogen source, implying ACC deaminase activity. The ACC deaminase activity of these four isolates, named ‘ACC1’, ‘ACC2’, ‘ACC3’ and ‘ACC4’, was further quantified in terms of α-ketobutyrate production.

#### 2.8.2. Quantitative Estimation of ACC Deaminase Activity

The ACC deaminase activity of isolates was assessed by α-ketobutyrate production via the catalyzation of the deamination reaction of the sole nitrogen source, ACC in DF minimal salt broth media at 540 nm. 

**Figure 8:**
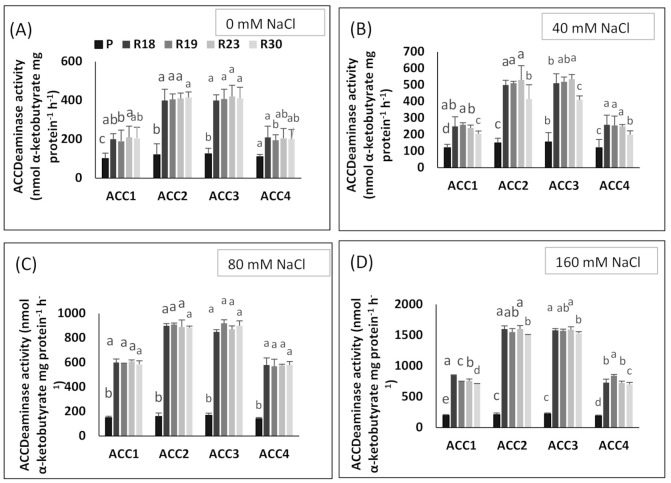
Quantification of ACC deaminase activity (nmol α-ketobutyrate mg protein^−1^ h^−1^) in response to NaCl treatments of four isolates from parent and regenerant roots. 0 mM NaCl (**A**), 40 mM NaCl (**B**), 80 mM NaCl (**C**) and 160 mM NaCl (**D**). Values are means of five replicates ± SE (*n* = 5). Significant dissimilarities between treatments (*p* < 0.05) based on Tukey’s HSD test are shown by different lowercase letters.

The ACC deaminase activity of the tested isolates exhibited variation, ranging from 400 to 1600 nmol α-ketobutyrate per mg of cellular protein per hour (Figure 8A–D). 

The regenerants showed higher ACC deaminase activity in all bacterial strains than parental control in all salinity treatments (Figure 8A–D). The highest ACC deaminase activity was shown by bacterial strains ‘ACC2’ and ‘ACC3’ in 0 mM NaCl (ranging from 400 to 420 nmol α-ketobutyrate mg protein^−1^ h^−1^), in 40 mM NaCl (ranging from 410 to 535 nmol α-ketobutyrate mg protein^−1^ h^−1^), in 80 mM NaCl (ranging from 850 to 920 nmol α-ketobutyrate mg protein^−1^ h^−1^) and in 160 mM NaCl (ranging from 1500 to 1600 nmol α-ketobutyrate mg protein^−1^ h^−1^) (Figure 7A–D). The most intensive enzymatic activity of ACC deaminase produced by ‘ACC2’ and ‘ACC3’, i.e., conversion of nitrogen source ACC into α-ketobutyrate, was confirmed according to [43].

#### 2.8.3. Quantification of Produced Indole Acetic Acid (Exp 2)

**Figure 9:**
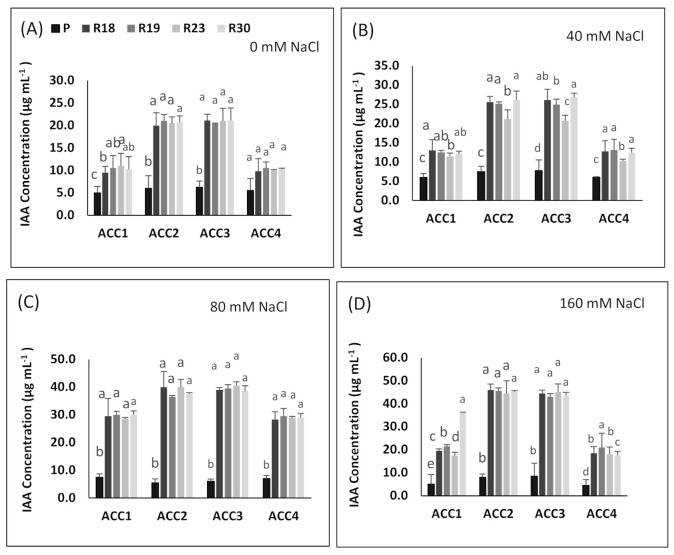
Quantification of IAA production (nmol α-ketobutyrate mg protein^−1^ h^−1^) in response to NaCl treatments of four isolates from parental line and regenerated plant roots. 0 mM NaCl (**A**), 40 mM NaCl (**B**), 80 mM NaCl (**C**) and 160 mM NaCl (**D**). Values are means of five replicates ± SE (*n* = 5). Significant dissimilarities between treatments (*p* < 0.05) based on Tukey’s HSD test are shown by different lowercase letters.

The quantification of IAA production by four ACC deaminase-producing isolates was achieved at 530 nm through supplementation of L-tryptophan to the growth media. The results are shown in Figure 9.

The accumulation of IAA was significantly more enhanced in regenerants than in the parental line in all treatments for all isolates. The highest IAA production was reached for the regenerated plants by ‘ACC2’ for 80 mM NaCl (ranging from 36.5 µg/mL to 40.1 µg/mL) and 160 mM NaCl (ranging from 44.5 µg/mL to 46 µg/mL for 160 mM NaCl) and by ‘ACC3’ for 80 mM NaCl (ranging from 38.5 µg/mL to 40.5 µg/mL) and 160 mM NaCl (ranging from 43 µg/mL to 45.1 µg/mL for 160 mM NaCl) (Figure 9C,D).

**Figure 10:**
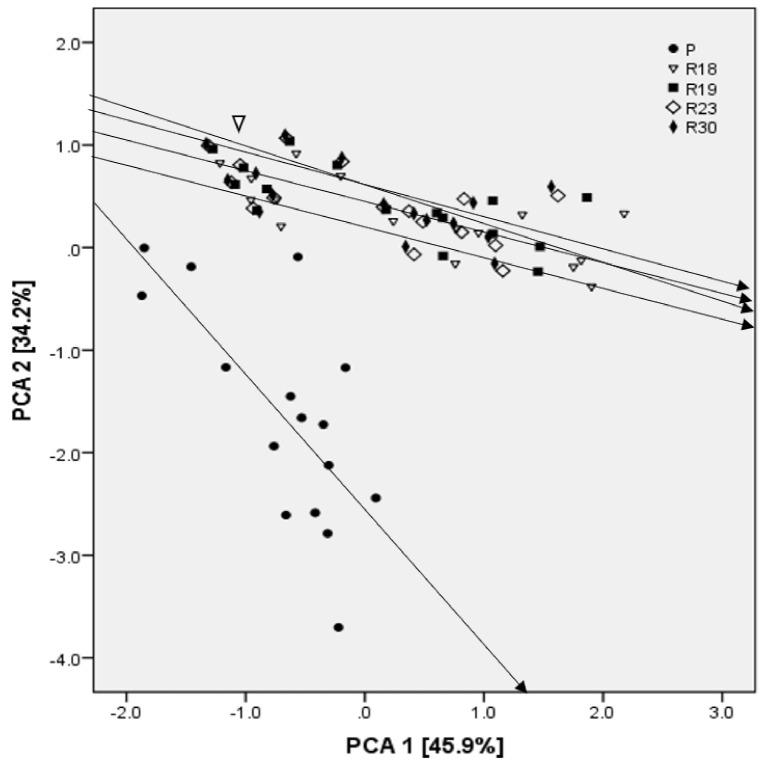
Principal component analysis (PCA) of starch, chlorophyll, proline, MDA, PSII, qp, Fv/Fm, ψ_l_ FW, DW, TWC, Na/K ratio in leaves, POD-L, APX-L, CAT-L, POD-R, APX-R and CAT-R of control parental and regenerated plants grown for 30 days under saline stress. PCA1 is positively correlated with CAT-R, POD-R, APX-R, APX-L, CAT-L, POD-L, Na/K-L, MDA and proline and negatively with PSII, qp, Fv/Fm, ψ_l_, TWC, FW and DW. PCA2 is positively correlated with CAT-R, POD-R, APX-R, APX-L, CAT-L, POD-L, Fv/Fm, TWC, FW, DW and chlorophyll and negatively with ψ_l_, MDA, starch and proline. Each data point represents the mean of four replicates. Arrows indicate the increasing salt stress level (●: P; ∇: R18; ■: R19; ◊: R23; ♦: R30).

A scores scatter plot of the first two PCAs (explaining 63.5% of the variation) shows a clear distinction between the regenerated plants and parent after 30 DSS (Figure 10). The loadings that had a positive correlation with PCA1 (45.9%) were CAT-R, POD-R, APX-R, APX-L, CAT-L, POD-L, Na/K-L, MDA and proline, and that with PCA2 (34.2%) were CAT-R, POD-R, APX-R, APX-L, CAT-L, POD-L, Fv/Fm, TWC, FW, DW and chlorophyll. The loadings of PSII, qp, Fv/Fm, ψ_l_, TWC, FW and DW had a negative correlation with PCA1. The loadings of ψ_l_, MDA, starch and proline had a negative correlation with PCA2. For regenerated and parental lines, the scores of the PCA moved to lower FW, DW, TWC, chlorophyll, qp, Fv/Fm, PSII and ψ_l_ and higher MDA, proline, starch, Na/K ratio in leaves, APX-L, CAT-L, POD-L, POD-R, APX-R and CAT-R values under increasing NaCl concentration. In parental line P, control and salt-stressed plants were highly separated along PCA1 in comparison to somaclones ‘R18’, ‘R19’, ‘R23’ and ‘R30’ (Figure 10). Control and salt-stressed plants showed a clear separation along PCA2 in ‘P’. 

## 3. Discussion

Salinity is still considered the most important abiotic stress threatening agriculture [58]. Somaclonal variation, a chromosomal/epigenetic rearrangement, can be used in breeding for salt tolerance. A phenotypic evaluation based on morphological, physiological and metabolic traits is indispensable to confirm stable mutations. Therefore, we evaluated the stability of salt tolerance in the progeny of four selected eggplant somaclonal variants (‘R18’, ‘R19’, ‘R23’ and ‘R30’) by assessing a number of agronomic, physiological and biochemical parameters at different developmental stages. 

The germination of seeds is the most vulnerable stage of development; therefore, the germinating seeds of most plant species do not tolerate salt. This has already been demonstrated by numerous vegetables such as lettuce [59] and beans [60]. The germination parameters (GP, MGT and MDG) were clearly affected by salinity. The seeds derived from the regenerants germinated 1–2 days slower than the control seeds, depending on the line (Figure 1). The salt limited water uptake by the seeds due to the lower osmotic potential of the germination medium, which contributed to an osmotically enforced “dormancy”. We propose the following explanation. Under salt stress, seeds develop an effective defense mechanism that prevents germination until more optimal conditions arise, such as rain in field conditions [61].

It should be noted that in our experiment, the germination of the control seeds decreased significantly at 80 mM NaCl. The same finding was reported for eggplant, in which a strong inhibition of germination at 100 mM NaCl was observed [62]. Moreover, the germination of susceptible tomato cultivars decreased strongly at 80 mM and drastically at 190 mM NaCl [16]. In addition, previous work demonstrated that the germination of sensitive lettuce varieties was substantially decreased at 100 and 150 mM of NaCl.

Similar results, describing a drastic decrease in seed germination, were found in tomato at 100 mM NaCl and in red beet, bell pepper, melon and broccoli at 150 mM NaCl [15]. The reduced MDT and MDG of our control seeds at 40 mM NaCl confirmed the salt sensitivity of eggplant. For comparison, decreases in MGT and MDG were described in Atriplex patula at 34 mM NaCl [63] and in Phaseolus at 120 mM NaCl [61]. According to [16], tomato seeds showed a crucial requirement of 50% more days to ensure germination at 80 mM NaCl than in a free salt medium and of almost 100% more days at a higher salt concentration (190 mM).

The salt sensitivity of eggplant during germination was confirmed at further growth stages. The decrease in growth parameters (shoot height, number of leaves, FW and DW) was more pronounced for ‘P’ compared to ‘R18’, ‘R19’, ‘R23’ and ‘R30’ (Table 1 and Table 2). Indeed, refs. [17,62] highlighted a growth reduction in eggplant under saline conditions at 100 mM NaCl. Exposing plants to saline conditions significantly reduced water and nutrient uptake during the initial phase. This was followed by excessive Na+ accumulation, leading to nutritional imbalances and water deficit during a second phase [30,64,65]. Ionic toxicity and osmotic adverse effects were associated during the onset and continued persistence of salt stress [18,66,67].

Excessive salinity causes stomata closure, which limits CO_2_ uptake, contributing to the dysfunction of the photosynthetic apparatus and the decrease in growth [30,68,69]. The effect of stomatal closure on the efficiency of photosynthesis is proportional to the amplitude of the reduction in leaf CO_2_ partial pressure (reviewed by [70]). Moreover, the significant amount of accumulated NaCl in chloroplasts induces the deterioration of PSII, which contributes to photodamage and affects photosynthetic efficiency [71,72,73,74]. 

To verify the impact of increasing salinity on the photochemistry of PSII, we performed Chl a fluorescence measurement in salt-tolerant eggplant mutants selected in vitro. Our study shows that increasing salinity has no significant effect on Fv/Fm in all regenerants after 30 days of salt stress. Similar findings have been reported for salt-tolerant tomato [36] and wheat [75]. The small reduction in Fv/Fm could be caused by the downregulation of PSII rather than photodamage to PSII [76,77].

The study of ΦPSII reflects PSII activity and adaptation [78]. It is clear that the decrease in growth is strongly related to the reduction in ΦPSII under saline conditions. Increasing salt concentration generated a significant decrease in control plants but a non-significant decrease in regenerants, due to the disruption of electron transport via PSII. These results are consistent with previous studies on salt-tolerant species reported by [79] in oaks, [36] in tomatoes, [80] in soybeans, [73] in roquettet and [74] in gingko. It is clear that the reduction in electron transport rate was less pronounced in the regenerants, demonstrating that they tolerate higher salt content than the parental control.

Similar to ΦPSII, increasing salt stress also contributed to lower qp in ‘P’, ‘R18’, ‘R19’, ‘R23’ and ‘R30’. However, the qp decrease was less pronounced in regenerants than in the parental control. As stated by [81], qp reflects the capacity of PSII in reducing the primary electron acceptor QA under salt conditions and indicates the number of photons used by photochemical reactions per number of assimilated photons. Our results show that ‘R18’, ‘R19’, ‘R23’ and ‘R30’ showed higher PSII reaction center activity combined with better efficiency in converting light energy into chemical energy than ‘P’ 

As highlighted by [77], photochemical quenching plays a major role in preserving the photosynthetic apparatus via shifting electrons to O_2_ under abiotic stress. Stomata closure contributes to a limitation of transpiration rates [36] and a reduction in assimilation efficiency [82] with increasing salinity. Ribulose bisphosphate carboxylase/oxygenase (Rubisco) is the main plant enzyme involved in the first phase of the C3 photosynthetic carbon reduction cycle [83]. During stomata closure, oxygenation of ribulose-1,5 bisphosphate in C3 plants replaces carboxylation [84,85,86,87].

Photosystem II is very vulnerable to salt stress. The absence of a significant effect of salinity on ΦPSII in all regenerated plants can be explained by the presence of an effective electron sink. Interestingly, the plants manage to maintain an effective conversion system of excitation energy via the downregulation of PSII RCs under salt condition [88]. Photoreduction could occur on the acceptor side of PSI in the Mehler reaction [89], which generates a pH gradient of the thylakoid membranes and produces a thermal loss of abundant excitation energy [90].

The decline in the non-photochemical energy loss is likely the main mechanism used to cope with photodamage [91]. The parental control exhibited a prominent rise in NPQ after 30 days of imposition of salt stress. 

It is clear that the increase in the NPQ of ‘P’ contributed greatly to counteract the decrease in Fv/Fm. The increased NPQ dissipates some of the abundant excitation energy at the expense of photochemical utilization [36,92], leading to a downregulation of PSII to avoid over-reduction in the primary electron acceptor QA. This reaction indicates a protective process to prevent photodamage in the photosynthetic machinery [76]. 

Under salinity conditions, foliar water potential and osmotic potential/EC changes are interrelated. It is obvious that the capacity of plants in maintaining a stable water potential difference between leaves and nutrient solution is of immense importance in order to impede desiccation. Salt stress decreased leaf turgor and atmospheric vapor pressure [93].

In [5], it was stated that the reduction in foliar water potential is associated with a linear decline in osmotic potential, thus leading to a fast diminishment in turgor potential. 

The regenerated plant succeeded in maintaining ψ_l_ and ψ_π_ at relatively stable levels under increasing salinity levels, while the parental control failed to do so. It is evident that the decrease in ψ_l_ in ‘P’ occurred simultaneously with the reduction in ψ_π_, thus keeping the same turgor status as the control. The aggravated decline in foliar ψ_π_ in parental controls might be explained by a substantial accumulation of Na^+^ and Cl^−^ (Table 7), considerably lowering water uptake, as reflected by the reduced TWC (Table 4), which contributed to the amplification of internal vacuole concentration. 

Many previous reports stated that salt stress reduced shoot and root growth, fresh and dry weight, plant height and yield characteristics [94,95].

The authors in [30] emphasized that a decline in growth appears in two steps: a fast reaction to osmotic stress is followed by a slower reaction induced by Na accumulation in foliar tissue. In our experiment, the regenerants exhibited higher salt tolerance than the parental control. This was evident through higher shoot biomass in ‘R18’, ‘R19’, ‘R23’ and ‘R30’ when compared to ‘P’. Our results agree with earlier reports indicating that the ability of regenerants in maintaining stable shoot biomass might be due to their capacity to cope with salinity at the cellular level through a mechanism of exclusion/avoidance [52,96,97]. While the excessive accumulation of salt in old foliar cell tissue continues, an attained toxic level decreases their ability to overcome salt influx (first phase). Then, an ionic specific response of the plant to salinity starts (second phase) [30].

The foliar tissue water content (TWC) represents an efficient stress indicator. All regenerated plants succeeded in maintaining higher and quite stable TWC compared to the parental control regardless of the salt concentration. Our findings indicate the ability of ‘R18’, ‘R19’, ‘R23’ and ‘R30’ in maintaining enhanced turgor and cell expansion using a performant plasmolytic process in order to overcome salt stress [97,98]. To cope with salinity, plants adopt an osmoregulatory strategy through the accumulation of organic low molecular weight solutes, enabling plants to maintain their plant–water status and positive carbon balance [18,99,100,101,102]. 

Proline catabolism is upregulated under increasing salinity concentrations by impeding its oxidation, which contributes to its substantial accumulation. Our results show that proline rose under salinity conditions in both parental control and regenerated plants. However, proline accumulation increase was more pronounced in ‘P’ than in ‘R18’, ‘R19’, ‘R23’ and ‘R30’. In the current work, it is noteworthy that the enhancement of proline accumulation seems to be induced by the metabolic damage generated by salt stress rather than appearing as a tolerance feature [103,104].

Saline conditions contribute to oxidative stress [105]. A high accumulation of reactive oxygen species leads to oxidative damage and engenders the membrane lipid peroxidation, thus affecting membrane fluidity and selectivity [101,106]. Membranes represent the most vulnerable cell organelles to stress-induced damage, and the extent of damage is considered an efficient tool for evaluating salt tolerance [105,107,108]. An apparent difference was detected between parental controls and regenerated plants in terms of lipid peroxidation under salt stress. ‘R18’, ‘R19’, ‘R23’ and ‘R30’ have the capacity of keeping lower levels of membrane peroxidation, indicating their superior capability for cellular protection to overcome the oxidative impairment caused by applied salt stress [109,110,111]. 

The increase in carbohydrates as a key factor involved in osmotic adjustment was stated previously by many authors [101,112]. These organic solutes provide the plant the ability to alleviate desiccation through improving its effectiveness in maintaining an osmotic balance in cell tissue [113]. Carbohydrates also function as the buffer of cellular redox potential, preventing cell dehydration by preserving the membrane and protein structural stability and providing enough energy sources under severe stress [114]. To overcome salinity-induced damage, plants adopt a process of compatible solutes accumulation, including amino acids, sugars and/or other composites [115]. 

Our results indicate that regenerated plants exhibited the ability to overcome a long and increasing salt stress, while they were successful in maintaining better growth by maintaining a higher chlorophyll content under salt stress conditions. In contrast, a high salinity level led to a chlorophyll content reduction in the parental control line, which contributed to an early leaf senescence and plant withering. 

As emphasized by [116], salinity contributes to K deficiency, thus reducing chlorophyll content and affecting photosynthetic apparatus functioning. Furthermore, salt-induced oxidative stress can also decrease the chlorophyll content [117,118]. Our experiments showed that the significant decline in the chlorophyll content was only limited to parental control, whereas no significant change was found in all the regenerated plants. 

This is in line with earlier reports [52,119,120,121] where rising salinity acted gravely on many processes; for example, water content, transpiration rate and leaf stomatal conductance in several crops. The negative effect of salt stress generated photosynthetic machinery damage through chloroplast dysfunction and chlorophyll depletion. 

As stated by [122], the availability of specific K^+^ and Ca^2+^ contents is crucially needed for preserving the integrity and functioning of cell membranes. The rise of Na^+^ and Cl^−^ content is considered as an osmoregulation process adopted by plants under salinity condition, while a decline in K^+^ and Ca^2+^ occurred in stressed plants [62].

In the present work, rising salinity level engendered an increase in foliar Na and Cl concentration associated with a reduction in K^+^, Ca^2+^, Mg^2+^ and NO_3_^−^ in the parental control and in all regenerated plants. However, all the regenerated plants exhibited reduced leaf concentration of Na^+^ and Cl^−^ and succeeded in keeping a more enhanced accumulation of K^+^, Ca^2+^ and Mg^2+^ compared to the parental control. This is consistent with earlier reports obtained by [17,18] for salt-tolerant eggplant lines.

It is worth noting that the parental control line showed an accentuated reduction in K content. K deficiency is mainly due to the strong competition between the absorbed NaCl and specifically K uptake [18,66]. Therefore, the rise in Na/K and Na/Ca ratios was more pronounced in ‘P’ than in ‘R18’, ‘R19’, ‘R23’ and ‘R30’. Consequently, salt tolerance or sensitivity in plants is deeply related to Na/K and Na/Ca ratios. As emphasized by [123,124], tolerant lines exhibit lower Na/K and Na/Ca ratios. 

To accomplish performant growth via normal cellular functioning, a reduced cytosol Na/K ratio is of immense importance in all plants. The imposition of salt stress and its continuity contributes to the inhibition of the K-specific transporters by Na, while competing with K uptake. Therefore, an excessive toxic concentration of Na is combined with a K deficiency [125,126]. Other salt-tolerant species are provided with an efficient osmotic potential regulation mechanism via preventing Na^+^ and Cl^−^ uptake and enhancing the absorption of other crucially required ions, such as K^+^ [123]. 

Moreover, to overcome the substantial amount of foliar Na, many glycophytes are provided with the mechanism of excluding Na and Cl^-^ from the cytosol through vacuole compartmentalization [127,128]. 

The high salt tolerance in the regenerants is closely related to a lower amount of accumulated salt ions in leaves. This might be explained by a more efficient mechanism of salt exclusion in ‘R18’, ‘R19’, ‘R23’ and ‘R30’ leaves (more tolerant) compared to ‘P’ (susceptible) [18,128].

A substantial salt ion accumulation contributes to the alteration of osmoregulation, the disturbance of membrane potential equilibrium and the loss of turgor [129]. Consequently, cell division and expansion are affected, thus causing deterioration in the plant biomass production process. 

A positive correlation between PTI and leaf Na^+^ concentration was evident. This result agrees with earlier findings. The authors of [130], working on Arabidopsis, and of [131], working on potato cultivars, emphasized that plants could be provided with mechanisms of Na^+^ tissue tolerance, such as the high accumulation of osmoregulants and intracellular compartmentation. The same hypothesis was adopted by [30] as an alternative tolerance process in glycophytes and obviously ‘R18’, ‘R19’, ‘R23’ and ‘R30’ acted by following the same strategy.

Under increasing salinity, the antioxidative machinery is upregulated in the regenerated plants. The effective protection developed by ‘R18’, ‘R19’, ‘R23’ and ‘R30’ against ROS in eggplant may be due to an efficient antioxidative system. These results are in line with many earlier reports [132,133]. 

As emphasized by [134,135], the strong oxidant H_2_O_2_ in peroxisomes is scavenged by CAT, and H_2_O is transformed into water and molecular O_2_. The enhanced activities of CAT and APX effectively decreased the rising amount of H_2_O_2_, thus leading to the amelioration of the cell membrane integrity and stability. In the parental control and the regenerated plants, CAT activity rose proportionally with the salt stress level. In ‘R18’, ‘R19’, ‘R23’ and ‘R30’, the most elevated salt concentration (160 mM NaCl) generated the highest CAT activity, thus leading to improved protection against ROS in all the regenerated plants. This result agrees with previous reports, stating that many salt-tolerant species exhibited higher CAT activity, such as maize [136], sesame [137], potato [138], melon [139] and wheat [140]. Several earlier authors reported that salinity increased APX activity in many salt-tolerant cultivars [106,137,141]. 

The susceptibility of the parental control could be due to the suppression of APX activity induced by rising salinity concentrations. This result is in concordance with previous reports dealing with Triticum aestivum [106], potato [138] and durum wheat [140]. APX is required to keep an acceptable intracellular amount of H_2_O_2_ in higher plants [142,143]. 

Our findings show that POD enzyme activities in parental control and regenerated plants were more enhanced with increasing salinity levels and were more elevated in ‘R18’, ‘R19’, ‘R23’ and ‘R30’ (salt-tolerant) than in ‘P’ (salt-sensitive). It is noteworthy that several previous studies stated that POD enzymes are associated with salt tolerance in melon [144], soybean [145] and wheat [140]. Consequently, POD enzymes contributed strongly to scavenging H_2_O_2_ in eggplant under salinity conditions. POD, which acts at both the intracellular and extracellular level, is an effective enzyme involved in the elimination of H_2_O_2_ [146]. 

The salt tolerance response of the regenerated plants could be engendered by its enhanced capacity in downregulating ROS production or by its improved ability to overcome ROS compared to the parental control. Our findings indicate that antioxidant enzyme machinery may have a strong contribution in scavenging H_2_O_2_ in eggplant. 

This is in line with earlier results reported by [147] working on tomatoes, [132] working on sugar beets, [85] working on *Populus euphratica*, [148] working on rice and [138] working on *Plantago*. 

ACC deaminase activity exhibited by PGPR has been proved to play a key role in improving growth and stress tolerance in plants under stressed conditions [149,150]. ACC deaminase, as a microbial enzyme, contributes to dissociating stress-induced ACC into ammonia and α-ketobutyrate, which produces ethylene that causes serious damage to the physiology, growth and development of plants [151,152]. In the present work, the salt tolerance response of the regenerated plants was associated with higher ACC deaminase activity in all bacterial strains and specifically in ‘ACC2’ and ‘ACC3’ when compared to the parental line showing lower values. The results of the current study agree with previous published works, reporting that bacteria provided by ACC deaminase activity are able to counteract the toxic impact of salt stress [153,154]. 

Under salt stress, PGPB contribute in enhancing IAA additional production, which may be useful in stimulating root growth, counteracting the inhibition impact of salinity on root and shoot growth and ameliorating plant physiological features [155]. 

In our study, all regenerants exhibited higher IAA accumulation in all isolates and specifically in ‘ACC2’ and ‘ACC3’ compared to the parental line in response to increasing salinity, which might be explained by their superior capacity to tolerate salt stress. This is consistent with previous work reported by [156,157,158]. 

In addition, the PCA analysis showed that Na^+^ was one of the criteria explaining the majority of dissimilarities among parental and regenerants lines, as well as the characteristics related to the antioxidant machinery, proline, PSII, MDA and the leaf water potential.

In this study, the progeny of somaclonal variants showed significant dissimilarities for several characters when compared to the parental line, and a higher level of salt tolerance. The statistical analysis of the whole set of data included in the current study confirmed the change in genetic stability. Regenerants with consistent performance at advanced generations are assumed to bear gene or chromosome variations, which seem to be stable and heritable. 

The success in applying somaclonal variation for salt tolerance depends on the rate and type of somaclonal variation obtained. Therefore, the low phenotypic variation among regenerants could be due to low physiological disturbance during tissue culture. Moreover, the identification of regenerants with putatively increased salt tolerance showed the positive impact of somaclonal variation in improving crop species for desired characters, besides the utility of culture-induced variation in genetic upgradation.

## 4. Conclusions

In this study, the stability of the salt tolerance of four previously obtained somaclonal variants was evaluated by characterizing the agronomic, physiological and biochemical parameters of their progeny. Seedlings from the parent plants and the regenerated plants were evaluated for salt tolerance under in vitro and realistic greenhouse conditions. Under the imposed salt stress, the results are consistent for germination kinetics, seedling growth and morphological and physiological responses, indicating that salt tolerance could be confirmed at both the germination and young plant stages. Metabolic homeostasis was less disturbed in the salt-tolerant plants, as indicated by the lower amount of MDA and loss of water content, despite the accumulation of proline and soluble carbohydrates. The salt tolerance of the regenerants was demonstrated by a stable primary photosynthetic apparatus and an effective antioxidant enzyme system. Two potential strains, ACC2 and ACC3, were found to possess other growth-promoting properties, such as the production of IAA. The efficiency of these strains in reducing salt stress and promoting plant growth was evident. The evaluation of the physiological and biochemical performance of the progeny of the in vitro regenerated plants demonstrates the great potential of callus culture for the development of variable genotypes and the selection of desirable traits in eggplant, such as genetically stable salt tolerance. From a more fundamental perspective, research on the level of field tolerance to other abiotic stresses of these genotypes selected for salt tolerance could be of interest. 

## 5. Materials and Methods

### 5.1. Plant Materials Setup of Experiments and Treatments

In previous work, an efficient regeneration protocol was developed to regenerate plants on salt-stress-tolerant calli lines derived from eggplant (‘Bonica F1’) leaf explants after a stepwise in vitro selection on media with increasing salinity (40, 80, 120 or 160 mM NaCl) [57]. Plants were regenerated on calli lines that could tolerate up to 120 mM NaCl. From the 32 plants tested in vitro, four regenerants with a higher number of leaves and root length were selected for further in vitro and in vivo evaluation. Seeds from parent plants (‘Bonica F1’) and from these four selected eggplant somaclones (R18, R19, R23 and R30) were harvested and used.

We performed two experiments: the first was an in vitro (germination and seedling growth) experiment (Exp 1), and the second was an in vivo (greenhouse) experiment (Exp 2).

#### 5.1.1. Experiment 1

Thiram-pretreated seeds of ‘Bonica’ (Vilmorin, France) were rinsed with 70% alcohol for some seconds and washed with distilled water. Then, they were surface sterilized for 20 min in a 5% HazTab solution (1,3,5 dichloro-triazine-trionedihydrate-dichlorosodium) and 0.02% Dreft (5–15% non-ionic surfactants, 15–30% anionic surfactants), followed by a solution of mercuric chloride (0.5%) for 10 min. After three rinses with sterile distilled water, the seeds were germinated on agar-solidified (0.8%) MS [159] medium with 3% (*w*/*v*) sucrose in 0.7 L glass vessels. The pH was adjusted to 5.8 with 1 N NaOH before autoclaving. NaCl was added to the medium at concentrations of 0 (control), 40, 80 and 160 mM.

Four seeds per vessel were used for every treatment and cultivar, in five replicates. The cultures were maintained in a growth chamber at 28 ± 2 °C, and a 16 h photoperiod regime was provided by cool-white fluorescent lamps with a photon flux density of 36 µmol m^–2^ s^–1^.

Each 24 h for one week, we investigated the germination (radicle to emergence) and the time to germinate. We determined the final germination percentage, the mean daily germination (MDG) and the mean germination time (MGT). The mean daily germination (MDG) and the mean germination time (MGT) were calculated according to [18]. 

After exposition to salt stress for 45 days (45 DSS), we randomly selected one seedling of each treatment. We determined seedling length, number of leaves, aerial fresh (FW) and dry weight (DW). The samples were then oven-dried at 70 °C for 24 h to determine the dry weight (DW). The tissue water content (TWC) was calculated using the formula: TWC = (FW − DW/FW).

A minimum of two seedlings per vessel were pooled, ground in liquid nitrogen and stored at −80 °C until metabolite analysis.

#### 5.1.2. Experiment 2

Germination took place in trays filled with peat and moistened with distilled water. The trays were placed in a controlled growth chamber at a photon flux density of 150 µmol m^−2^ s^−1^, a constant temperature of 25 °C and 70% relative humidity (RH). After 25 days, seedlings showing their second true leaf were transferred to 2-litre plastic pots filled with peat and kept in a heated greenhouse (36°50′ N, 10°11′ E) at the Department of Plant Physiology and Biotechnology, National Institute of Agricultural Research, Tunisia. For the next 36 days, each seedling was irrigated with 250 mL of Hoagland solution [160] at full strength. The parental control line and the 4 selected somaclones received the following salt stress treatments: 0 (control), 40, 80 and 160 mM NaCl. The control plants were irrigated with 250 mL of distilled water twice a week for 30 days, while the salt-stressed plants were irrigated with 250 mL of 40, 80 and 160 mM NaCl solution twice a week for 30 days. Twenty plants per line (5 plants/block) were subjected to each treatment. In our experiment, a randomized block design was applied with five replicates for each treatment and line.

### 5.2. Plant Growth and Water Status Measurements

After being exposed to salt stress for 30 days (30 DSS), we randomly selected eight plants of each treatment. We determined shoot and leaf fresh weight (FW). The samples were then oven-dried at 70 °C for 48 h to determine the dry weight (DW). The tissue water content (TWC) was calculated using the formula: TWC = (FW − DW/FW). The Scholander pressure chamber (model 1000, PMS Instrument Company, Albany, OR, USA) was used to determine the leaf water potential (ψ_midday_) of the youngest fully expanded leaves. We determined the leaf osmotic potential (ψ_π_) according to [161]. Measurements were performed in four repetitions. A plant tolerance index (PTI) was calculated based on the total fresh weight (FW) in salt-stressed plants per total FW in control plants.

### 5.3. Chl a Fluorescence Measurements

Chl *a* fluorescence measurements were performed in the dark- and light-adapted leaves with a portable fluorometer (PAM-2500, Walz, Effeltrich, Germany). After 30 min of dark adaptation, Fv/Fm was estimated as (Fm − F_0_)/Fm, where Fm (induced by a short pulse (0.6 s) of saturating light (3450 µmol m^–2^ s^–1^)) and F_0_ were the maximum and minimum fluorescence, respectively [78]. After 4 min of illumination with uninterrupted red, nonsaturating actinic light (447 µmol m^–2^ s^–1^) and saturating pulses every 25 s, the maximum (Fm’) and the constant-state (Fs) fluorescence signals were measured in the light-adapted leaves. Then, the actinic light was stopped and the far-red pulse was applied to obtain the minimal fluorescence after the PSI excitation (F_0_’). Φ_PSII_ was calculated as (Fm’ − Fs)/Fm’ and qp was estimated as (Fm’ − Fs)/(Fm’ − F_0_’) [162]. NPQ, which is proportional to the rate constant of the thermal energy dissipation, was calculated as (Fm − Fm’)/Fm’ [163]. The electron transport rate (ETR) was estimated as Φ_PSII_ × PAR × 0.84 × 0.5, where the absorbed photon energy (PAR) was assumed to be equally distributed between PSI and PSII, and 0.84 was the assumed light absorptance of the leaf. Measurements were applied on the youngest, fully developed leaf after 5, 10, 15, 20, and 30 d of salt stress (DSS) in four replicates.

### 5.4. Leaf Chlorophyll Content Measurements

For all plants and for all salt concentrations, we determined leaf chlorophyll content by means of a chlorophyll meter (Konica Minolta SPAD-502, Tokyo, Japan). We performed three SPAD measurements from three fully expanded leaves from the lower, middle and upper canopy. Thus, chlorophyll content of each plant was assayed as an average of nine SPAD readings per plant. Chlorophyll data were assessed on two dates: five and ten weeks after beginning salt stress imposition.

### 5.5. Metabolites Extraction and Analysis

After 30 days of salt stress, we reaped fully developed upper leaves (2 leaves/replicate in a bulked sample) between 12 h and 14 h from four plants, in each treatment (1 plant/block) and for each variety. We ground harvested leaf material in liquid nitrogen and stored it at −80 °C until analysis.

Soluble sugars were extracted by 80% ethanol at 70 °C for 10 min and further at 45 °C for 3 h, followed by centrifugation at 5000× *g* for 5 min [18]. HPLC was used to determine the sugars (Waters; CarboPac MA1 column with companion guard column, eluent: 50 mM NaOH, 22 °C).

The rest of the ethanol insoluble material was washed two times with ethanol 80% and the pellet residue was treated with HCl 1 M for 2 h at 95 °C for starch hydrolysis. Starch extraction and quantification were achieved according to [18] and based on the enzymatic reduction of NADP^+^.

We quantified proline according to [164]. In short, 500 mg of plant tissue was extracted with 3% (*w*/*v*) sulfosalicylic acid. The determination of proline was carried out using a calibration curve and expressed as µg proline g^−1^ FW [18].

Lipid peroxidation was investigated through the determination of malondialdehyde (MDA) [18,165]. In short, we extracted 1 g of leaf material in 80% ethanol. The determination of MDA was based on the reaction with thiobarbituric acid (TBA), and the absorbance was measured at λ = 440 nm, 532 nm and 600 nm by spectrophotometer (InfiniteM200 TECAN Group Ltd., Männedorf, Switzerland). Malondialdehyde (MDA) equivalents were estimated according to [165].

### 5.6. Enzymatic Assays

For protein and enzyme extractions, 0.5 g of leaf and root samples were homogenized with 50 mM potassium phosphate buffer (pH 7.8) containing 1 mM EDTA-2Na and 7% (*w*/*v*) polyvinylpolypyrrolidone (PVPP). The whole extraction procedure was carried out at 4 °C. A centrifugation of the homogenates was performed at 4 °C for 15 min at 130,009× *g*, and enzyme activity was measured using the supernatants. Protein was quantified as described by [166], utilizing bovine serum albumin as a standard.

According to [167], catalase (CAT) activity (EC 1.11.1.6) was assayed by the determination of the level of decomposition of H_2_O_2_ (ε = 2.3 mM^−1^ cm^−1^) at 240 nm. This activity was measured in a reaction mixture containing 1900 µL of potassium phosphate buffer (50 mM, pH 7.0 not containing EDTA), 100 µL sample and 1000 µL H_2_O_2_ (30 mM). CAT activity was expressed as µmol H_2_O_2_ decomposed min^−1^ mg^−1^ proteins.

Ascorbate peroxidase (APX) activity (EC 1.11.1.11) was determined according to [168]. The reaction mixture contained 50 mM of potassium phosphate buffer (pH 7.0), 4.4 µL ascorbate (1 mM) and 10 µL EDTA-2Na (0.5 M). Adding H_2_O_2_ started the reaction and ascorbate oxidation was determined at 290 nm for 1 min. Activity was quantified using the extinction coefficient, e = 2.8 mM^−1^ cm^−1^. Each sample was measured in three repetitions. Results are expressed as µmol oxidized ascorbate min^−1^ mg^−1^ proteins.

Guaiacol peroxidase (POD) activity (EC 1.11.1.7) was quantified according to [169]. The reaction solution included 100 µL of plant extract supplemented with 700 µL of 0.05 M phosphate buffer (pH 7.8) and 200 µL of guaiacol (25 mM). The reaction began by adding 100 µL of H_2_O_2_. The absorbance elevation generated by the oxidation of guaiacol to tetra guaiacol was recorded for 3 min at 436 nm. POD activity was estimated from the extinction coefficient, ε = 25.5 mM^−1^ cm^−1^. Results are expressed as µmol oxidized guaiacol min^−1^ mg^−1^ proteins.

### 5.7. Mineral Content

Leaf and root samples were harvested from four plants in every treatment (1 plant/block) and for every cultivar, washed, oven-dried at 70 °C for 48 h and finally grounded. The determination of P, K, Ca, Mg, S and Na contents was performed by ICP-OES after dry-ashing at 550 °C. A potentiometric analysis using an ion-selective electrode (VWR, Leuven, Belgium) for chlorides was achieved.

### 5.8. ACC Deaminase Producing Bacteria Assay

#### 5.8.1. Collection of Rhizospheric Soil Sample

The root samples of Bonica and Black Beauty were collected from 6 pots for each cultivar in every treatment during May and June 2021 in a heated glasshouse (36°50′ N, 10°11′ E) at the Department of Plant physiology and Biotechnology, National Institute of Agronomic Research, Tunisia. Four plants/cultivar/treatment were collected from each pot and brought to the laboratory in closed plastic bags for further analysis. The roots were separated from each plant, crushed and mixed together to form one composite pool of root sample.

#### 5.8.2. Isolation of Bacteria and Qualitative Estimation of ACC Deaminase Activity

The isolation of bacteria and the qualitative determination of ACC deaminase activity were achieved according to [149]. Briefly, the bacteria were isolated from the roots sample by serial dilution technique in Luria-Bertani (LB) medium. The morphologically different colonies were subjected to ACC deaminase activity screening on the sterile minimal DF (Dworkin and Foster) salts media corrected with 3 mM ACC as sole nitrogen source [170,171]. The inoculated plates were incubated at 28 °C during 3 days and growth was observed daily. The growing colonies were considered as ACC deaminase producers and were purified by sub culturing the isolates.

#### 5.8.3. Quantification of ACC Deaminase Activity

The quantitative determination of ACC deaminase activity was performed according to [171]. This method assesses the amount of α-ketobutyrate resulted of the cleavage of ACC by ACC deaminase. The ACC deaminase activity was expressed in mmol α-ketobutyrate mg protein^−1^ h^−1^.

#### 5.8.4. Indole Acetic Acid Production by Bacterial Isolates

According to [149] the bacterial strains were inoculated in LB medium corrected with 5 mM tryptophan and incubated in orbital shaker for 7 days at 28 °C at 200 rpm. The IAA quantification was achieved via the colorimetric method using Salkowski reagent (0.5 M FeCl_3_ + 70% perchloric acid). Development of red color (which indicates the formation of indolic compounds) with addition of Salkowski reagent and cell free culture supernatant (4:1) was measured by UV–vis spectrophotometer at 530 nm [172]. The concentration of IAA can be determined with a standard curve of pure indole-3-acetic acid (IAA, Hi-media) ranging between 0 and 100 mg mL^−1^.

### 5.9. Statistical Analysis

A completely random design was used in conducting all analyses. The detection of significant dissimilarities between treatments or varieties was completed using SPSS Statistics 21 after subjecting all collected data to a one-way analysis of variance (ANOVA). The comparison of means was performed using the Tukey’s multiple range test (*p* = 0.05). We performed the principal component analysis (PCA) on starch, chlorophyll, proline, MDA, PSII, qp, Fv/Fm, ψ_l_ FW, DW, TWC, Na/K ratio in leaves, POD, APX and CAT activity in leaves/roots of parental control and regenerated plants grown for 30 days under salt stress. PCAs with eigenvalues > 1, thus explaining more than a single parameter alone, were examined. For these principal components, a varimax rotation was applied to the resulting factor loading. According to [173], this rotation offers simpler factors, relating parameters essentially to one principal component axis. All statistical analyses were carried out employing SPSS 25 (IBM SPSS Statistics).

## Figures and Tables

**Table 1:** Effect of increasing NaCl concentration on PH, LN, FW, DW and TWC of eggplant seedlings after 45 days of growth. No data are given for 160 mM due to the high mortality for this salt concentration (Exp 1).

Cultivar	NaCl (mM)	LN	PH (cm)	FW (g)	DW (g)	TWC (%)
P	0	5.0 ± 0.1 ^a^	10.1 ± 0.5 ^a^	6.40 ± 0.14 ^a^	0.97 ± 0.01 ^a^	0.85 ± 0.0 ^a^
	40	3.8 ± 0.3 ^b^	9.30 ± 0.4 ^a^	2.20 ± 0.25 ^b^	0.51 ± 0.01 ^b^	0.77 ± 0.0 ^b^
	80	1.6 ± 0.2 ^c^	4.10 ± 0.2 ^b^	0.35 ± 0.09 ^c^	0.21 ± 0.01 ^c^	0.40 ± 0.0 ^b^
R18	0	5.0 ± 0.1 ^a^	9.30 ± 0.5 ^a^	5.50 ± 0.17 ^a^	0.99 ± 0.02 ^a^	0.82 ± 0.0 ^a^
	40	5.4 ± 0.2 ^a^	10.1 ± 0.4 ^a^	4.40 ± 0.21 ^b^	0.76 ± 0.03 ^b^	0.83 ± 0.0 ^a^
	80	4.1 ± 0.1 ^a^	4.10 ± 0.2 ^b^	3.70 ± 0.04 ^c^	0.64 ± 0.02 ^c^	0.83 ± 0.0 ^a^
R19	0	6.1 ± 0.1 ^a^	10.2 ± 0.5 ^a^	5.50 ± 0.16 ^a^	1.00 ± 0.02 ^a^	0.82 ± 0.0 ^a^
	40	6.3 ± 0.2 ^a^	11.1 ± 0.4 ^a^	4.20 ± 0.13 ^b^	0.76 ± 0.02 ^b^	0.82 ± 0.0 ^a^
	80	5.2 ± 0.1 ^a^	5.2 ± 0.2 ^b^	3.60 ± 0.11 ^c^	0.65 ± 0.03 ^c^	0.82 ± 0.0 ^a^
R23	0	5.0 ± 0.3 ^a^	10.7 ± 0.2 ^b^	6.10 ± 0.20 ^a^	1.10 ± 0.03 ^a^	0.82 ± 0.0 ^a^
	40	5.5 ± 0.2 ^a^	12.5 ± 0.3 ^a^	4.90 ± 0.14 ^b^	0.84 ± 0.01 ^b^	0.83 ± 0.0 ^a^
	80	4.8 ± 0.1 ^a^	7.00 ± 0.1 ^c^	3.80 ± 0.24 ^c^	0.66 ± 0.06 ^c^	0.83 ± 0.0 ^a^
R30	0	5.5 ± 0.2 ^a^	11.3 ± 0.4 ^a^	6.50 ± 0.18 ^a^	1.10 ± 0.03 ^a^	0.83 ± 0.0 ^a^
	40	6.1 ± 0.1 ^a^	11.9 ± 0.3 ^a^	5.00 ± 0.12 ^b^	0.84 ± 0.04 ^b^	0.83 ± 0.0 ^a^
	80	5.1 ± 0.3 ^a^	6.10 ± 0.1 ^b^	3.90 ± 0.19 ^c^	0.66 ± 0.07 ^c^	0.83 ± 0.0 ^a^

Values are means ± standard errors of four replicates. Significant dissimilarities between treatments (*p* ≤ 0.05) based on Tukey’s HSD test are shown by different lowercase letters within each salinity level (*n* = 5).

**Table 2:** Effect of increasing levels of NaCl on FW of parents and regenerants.

Lines	FW (g)			
	0 mM	40 mM	80 mM	160 mM
P	140.9 ± 3.2 b	107.9 ± 2.5 b	80.6 ± 3.2 b	37.8 ± 3.5 b
R18	215.5 ± 2.7 a	214.9 ± 3.1 a	200.4 ± 5.1 a	148.0 ± 2.1 a
R19	216.5 ± 2.1 a	215.5 ± 1.2 a	200.3 ± 2.0 a	148.6 ± 2.0 a
R23	217.5 ± 1.9 a	216.5 ± 0.9 a	202.3 ± 5.1 a	148.0 ± 2.1 a
R30	217.1 ± 4.2 a	215.5 ± 1.7 a	201 ± 3.6 a	150.0 ± 3.9 a

Values are means ± SE (*n* = 5). Significant dissimilarities between treatments (*p* ≤ 0.05) based on Tukey’s HSD test are shown by different lowercase letters within each salinity level.

**Table 3:** Effect of increasing levels of NaCl on DW of parents and regenerants.

Lines	Dry Weight (g)			
	0 mM	40 mM	80 mM	160 mM
P	25.7 ± 1.2 b	23.7 ± 1.5 b	20.3 ± 4.2 b	12.1 ± 4.5 b
R18	31 ± 0.7 a	30.9 ± 1.9 a	29.1 ± 3.1 a	22.2 ± 1.1 a
R19	31.5 ± 1.1 a	31.0 ± 2.1 a	29.4 ± 1.0 a	22.3 ± 3.1 a
R23	31.5 ± 2.9 a	31.0 ± 0.9 a	29.4 ± 3.1 a	22.2 ± 1.1 a
R30	33.2 ± 3.2 a	32.7 ± 1.2 a	31.1 ± 3.7 a	24.0 ± 3.9 a

Values are means ± SE (*n* = 5). Significant dissimilarities between treatments (*p* ≤ 0.05) based on Tukey’s HSD test are shown by different lowercase letters within each salinity level.

**Table 4:** Effect of increasing levels of NaCl on TWC of parents and regenerants.

Lines	TWC (g)			
	0 mM	40 mM	80 mM	160 mM
P	0.81 ± 0.9 b	0.78 ± 0.7 b	0.74 ± 3.2 b	0.68 ± 2.1 b
R18	0.85 ± 2.7 a	0.85 ± 1.2 a	0.85 ± 1.3 a	0.85 ± 1.1 a
R19	0.85 ± 2.1 a	0.85 ± 1.1 a	0.85 ± 0.9 a	0.85 ± 2.3 a
R23	0.85 ±1.5 a	0.85 ± 1.1 a	0.85 ± 1.3 a	0.85 ± 0.9 a
R30	0.84 ± 2.2 a	0.84 ± 0.9 a	0.84 ± 2.5 a	0.84 ± 2.9 a

Values are means ± SE (*n* = 5). Significant dissimilarities between treatments (*p* ≤ 0.05) based on Tukey’s HSD test are shown by different lowercase letters within each salinity level.

**Table 5:** Yield (fruit number per plant and mean fresh weight per fruit) of parents and regenerants subjected to increasing salt stress level for five weeks and harvested 45 days after flowering.

Parameter	NaCl (mM)	P	R18	R19	R23	R30
Fruit	0	7 ± 1.7 b	11 ± 1.5 a	11 ± 1.4 a	13 ± 1.3 a	11 ± 1.3 a
number	80	4 ± 1.2 b	13 ± 0.4 a	13 ± 1.1 a	14 ± 2.2 a	13 ± 1.2 a
	120	1 ± 1.6 b	11 ± 1.3 a	11 ± 1.3 a	12 ± 2.5 a	10 ± 1.2 a
	0	183 ± 1.5 b	254 ± 3.2 a	240 ± 1.5 a	243 ± 2.6 a	222 ± 2.3 a
Fruit	80	156 ± 3.1 b	263 ± 2.7 a	247 ± 2.6 a	258 ± 2.4 a	230 ± 2.2 b
weight	120	82 ± 1.2 b	182 ± 1.5 a	190 ± 2.1 a	198 ± 1.9 a	180 ± 0.5 a

Values are means ± SE (*n* = 5). Significant dissimilarities between treatments (*p* ≤ 0.05) based on Tukey’s HSD test are shown by different lowercase letters within each salinity level.

**Table 6:** Chlorophyll data average of parents and regenerants subjected to increasing salt stress levels during five weeks in the greenhouse.

Lines	Average Chlorphyll Content (µg/cm^2^)			
	0 mM	40 mM	80 mM	160 mM
P	33.8 ± 4.2 b	32.6 ± 3.2 b	25.1 ± 3.5 b	17.5 ± 2.3 b
R18	45.9 ± 2.9 a	54.4 ± 5.1 a	50.7 ± 2.1 a	46.9 ± 2.1 a
R19	48.1 ± 2.2 a	55.0 ± 2.0 a	52.3 ± 2.0 a	49.5 ±1.8 a
R23	51.5 ±2.9 a	56.9 ± 5.1 a	56.2 ± 2.1 a	55.4 ± 3.3 a
R30	53.1 ± 3.2 a	58.6 ± 3.6 a	57.4 ± 3.9 a	56.1 ± 2.4 a

Values are means ± SE (*n* = 5). Significant dissimilarities between treatments (*p* ≤ 0.05) based on Tukey’s HSD test are shown by different lowercase letters within each salinity level.

**Table 7:** Average chlorophyll data of parents and regenerants subjected to increasing salt stress levels during ten weeks in the greenhouse.

Lines	Average Chlorphyll Content (µg/cm^2^)			
	0 mM	40 mM	80 mM	160 mM
P	61.8 ± 3.4 b	58.4 ± 3.1 b	45.2 ± 3.7 b	40.8 ± 3.7 b
R18	79.8 ± 5.3 a	85.5 ± 1.2 a	89.7 ± 3.6 a	84.3 ± 1.9 a
R19	80.5 ± 4.6 a	87.7 ± 3.6 a	91.9 ± 4.1 a	85.2 ± 2.2 a
R23	77.4 ± 4.4 a	67.9 ± 2.9 b	82.1 ± 4.6 a	66.1 ± 3.1 a
R30	79.0 ± 4.6 a	69.5 ± 4.0 b	83.7 ± 3.2 a	65.7 ± 1.3 a

Values are means ± SE (*n* = 5). Significant dissimilarities between treatments (*p* ≤ 0.05) based on Tukey’s HSD test are shown by different lowercase letters within each salinity level.

**Table 8:** The effect of NaCl salinity on K, Ca, Mg, Na, P, NO_3_^−^ and Cl^−^ accumulation and on Na/K and Na/Ca ratios in control parent and regenerant leaves.

Cultivar	NaCl	K^+^	Ca^2+^	Mg^2+^	Na^+^	P	NO_3_^−^	Cl^−^	Na/K	Na/Ca
	(mM)	(g/100 g DS)								
P	0	6.4 ± 0.2 a	3.1 ± 0.4 a	0.40 ± 0.01 a	1.14 ± 0.2 c	0.93 ± 0.3 a	4.2 ± 0.6 a	2.5 ± 0.2 c	0.17 ± 0.12 c	0.3 ± 0.15 c
	40	5.8 ± 0.1 a	2.9 ± 0.2 a	0.38 ± 0.01 ab	2.66 ± 0.2 b	0.83 ± 0.4 ab	5.5 ± 0.7 a	3.6 ± 0.6 c	0.45 ± 0.08 c	0.89 ± 0.09 c
	80	4.5 ± 0.3 b	2.4 ± 0.1 ab	0.25 ± 0.03 b	5.23 ± 0 a	0.81 ± 0.1 ab	3.7 ± 0.2 b	7.1 ± 0.1 b	1.15 ± 0.04 b	2.11 ± 0.05 b
	160	3.7 ± 0.4 b	1.9 ± 0.2 b	0.22 ± 0.01 b	6.42 ± 1.2 a	0.69 ± 0.1 b	1.3 ± 0.4 c	11.4 ± 0.2 a	1.73 ± 0.02 a	3.28 ± 0.30 a
R18	0	5.8 ± 0.2 a	3.6 ± 0.2 a	0.52 ± 0.04 a	0.91 ± 0.2 b	0.77 ± 0.2 a	1.9 ± 0.2 a	3.2 ± 0.8 c	0.15 ± 0.08 c	0.25 ± 0.07 c
	40	5.4 ± 0.2 a	3.1 ± 0.2 a	0.50 ± 0.01 a	1.96 ± 0.2 ab	0.75 ± 0.2 a	1.3 ± 0.5 a	5.5 ± 0.6 bc	0.36 ± 0.06 bc	0.63 ± 0.08 bc
	80	5.3 ± 0.3 a	3.0 ± 0.3 a	0.48 ± 0.02 a	2.17 ± 0.1 a	0.69 ± 0.2 a	1.6 ± 0.2 a	7.1 ± 0.3 ab	0.40 ± 0.04 ab	0.72 ± 0.08 ab
	160	4.4 ± 0.1 b	2.9 ± 0.1 a	0.46 ± 0.01 a	2.27 ± 0.1 a	0.65 ± 0.1 a	0.73 ± 0.2 a	8.9 ± 0.3 a	0.51 ± 0.02 a	0.78 ± 0.02 a
R19	0	5.9 ± 0.3 a	3.7 ± 0.1 a	0.53 ± 0.02 a	0.92 ± 0.3 b	0.79 ± 0.5 a	1.9 ± 0.3 a	3.2 ± 0.1 d	0.15 ± 0.02 c	0.25 ± 0.02 b
	40	5.5 ± 0.1 a	3.2 ± 0.4 a	0.51 ± 0.02 a	2.00 ± 0.2 ab	0.77 ± 0.2 a	1.3 ± 0.3 a	5.6 ± 0.4 c	0.36 ± 0.05 bc	0.62 ± 0.06 ab
	80	5.4 ± 0.4 a	3.0 ± 0.2 a	0.49 ± 0.01 a	2.18 ± 0.1 a	0.71 ± 0.2 a	1.6 ± 0.1 a	7.3 ± 0.2 b	0.39 ± 0.05 ab	0.70 ± 0.04 a
	160	4.5 ± 0.2 b	2.9 ± 0.2 a	0.46 ± 0.01 a	2.28 ± 1.1 a	0.66 ± 0.1 a	0.75 ± 0.5 a	9.1 ± 0.1 a	0.50 ± 0.03 a	0.76 ± 0.12 a
R23	0	7	3.2 ± 0.1 a	0.51 ± 0.03 a	0.84 ± 0.3 b	0.92 ± 0.3 a	3.5 ± 0.7 a	2.8 ± 0.8 c	0.14 ± 0.06 c	0.26 ± 0.16 b
	40	5.8 ± 0.1 a	3.1 ± 0.1 a	0.49 ± 0.04 ab	1.8 ± 0.2 ab	0.87 ± 0.1 a	2.7 ± 0.2 ab	4.6 ± 0.4 b	0.31 ± 0.08 bc	0.58 ± 0.06 ab
	80	5.7 ± 0.3 ab	3.0 ±0.3 a	0.46 ± 0.03 ab	2.2 ± 0.1 a	0.84 ± 0.1 a	1.9 ± 0.4 bc	7.1 ± 0.4 a	0.38 ± 0.04 ab	0.73 ± 0.08 a
	160	4.2± 0.2 b	3.0 ± 0.2 a	0.44 ± 0.01 b	2.6 ± 0.1 a	0.84 ± 0.2 a	1.0 ± 0.1 c	8.3 ± 0.3 a	0.61 ± 0.04 a	0.86 ± 0.04 a
R30	0	6.2 ± 0.2 a	3.4 ± 0.5 a	0.53 ± 0.02 a	0.83 ± 1.2 b	0.94 ± 0.2 a	3.5 ± 0.5 a	2.7 ± 0.1 c	0.13 ± 0.05 c	0.24 ± 0.12 b
	40	6.0 ± 0.2 a	3.3 ± 0.3 a	0.51 ± 0.04 a	1.74 ± 0.2 ab	0.89 ± 0.4 a	2.6 ± 0.5 ab	4.6 ± 0.4 b	0.29 ± 0.05 bc	0.52 ± 0.09 ab
	80	5.9 ± 0.1 ab	3.2 ± 0.2 a	0.49 ± 0.02 a	2.11 ± 0.4 a	0.85 ± 0.4 a	1.8 ± 0.2 bc	7.2 ± 0.5 a	0.35 ± 0.07 ab	0.65 ± 0.13 a
	160	4.6 ± 0.3 b	3.2 ± 0.2 a	0.47 ± 0.01 a	2.52 ± 0.3 a	0.87 ± 0.1 a	0.92 ± 0.2 c	8.4 ± 0.2 a	0.54 ± 0.02 a	0.78 ± 0.07 a

Means followed by the same lowercase letter within each column and cultivar are not significantly different at *p* = 0.05 according to Tukey’s HSD test (*n* = 5).

## Data Availability

No new data were created or analyzed in this study. Data sharing is not applicable to this article.
